# Association of NDRG4 gene methylation in peripheral blood leukocytes with gastric cancer risk, chemotherapy efficacy and prognosis

**DOI:** 10.3389/fonc.2026.1778070

**Published:** 2026-04-27

**Authors:** Zhan Li, Yuan Chen, Dingtao Hu, Fengling Luo, Hao Gao, Yuxin Wang, Yaping Feng, Yue Li, Yingying Yu, Yuqin Tang, Mingyi Zhang, Yanfeng Zou, Fang Wang

**Affiliations:** 1 Department of Oncology, The First Affiliated Hospital of Anhui Medical University, Hefei, Anhui, China; 2 Department of Epidemiology and Biostatistics, School of Public Health, Anhui Medical University, Hefei, Anhui, China

**Keywords:** gastric cancer, methylation, NDRG4 gene, peripheral blood leukocytes, risk

## Abstract

**Objective:**

To analyze the functions of NDRG family genes in gastric cancer (GC) utilizing bioinformatics. Furthermore, the association between the methylation of NDRG4 gene in peripheral blood leukocytes (PBLs) and GC risk, chemotherapy efficacy, and prognosis was analyzed.

**Methods:**

Using TCGA and GEO databases, the study analyzed the expression, immune related signatures, methylation patterns and prognostic of NDRG family genes. Two-phase case-control study (310 GC patients and 300 controls) was conducted to investigate the association between the methylation of NDRG4 gene and GC risk. In addition, this study explored the association between methylation of NDRG4 gene and the therapeutic effect and prognosis of GC. The expression level of NDRG4 gene was compared between the hypermethylation and hypomethylation groups. The single nucleotide polymorphism (SNP) rs7202037 genotypes of 280 GC patients were detected, and the methylation level differences among rs7202037 genotypes were compared.

**Results:**

Bioinformatics analysis showed that the expression of NDRG family genes was related to immunity and methylation (*P* < 0.05). Two-phase case-control study found that the methylation of NDRG4-chr16:58497239 (*OR* = 2.536, 95%*CI* = 1.514-4.248, *P*_BH_=0.004) and NDRG4-chr16:58497262 (*OR* = 1.910, 95%*CI* = 1.223-2.981, *P*_BH_=0.020) sites were correlated with GC risk. The two CpG sites was also associated with chemotherapy efficacy (*P*_BH_<0.05). The methylation of NDRG4-chr16:58497239 was correlated with progression free survival (PFS) of GC patients (*P*_BH_=0.016). Compared with the hypermethylation group, NDRG4 was expressed higher in hypomethylation group (*P* = 0.019). Methylation levels differed among different rs7202037 genotypes (*P* < 0.05).

**Conclusions:**

The methylation of NDRG4 gene in PBLs was associated with GC risk, chemotherapy efficacy and prognosis. The rs7202037 variation could affect the methylation levels of NDRG4 gene. These findings provided new directions for exploring the pathogenesis of GC and screening non-invasive marker.

## 1 Introduction

Gastric cancer (GC) is the second leading cause of cancer deaths worldwide (1). Globally, there were nearly one million new cases each year, resulting in over 650,000 deaths (2). Early detection is of great significance for reducing the disease burden and mortality rate of GC. However, many patients have already reached the advanced stage when they are diagnosed (3). Endoscopy is helpful for the early diagnosis of GC, but it is invasive and expensive (4). At present, blood-based biomarkers have great potential in non-invasive tumor detection (5). GC is mainly affected by genetic, epigenetic, and environmental factors (6). DNA methylation plays an important role in the occurrence and development of cancer. In various cancers, abnormal DNA methylation often induces tumor suppressor gene silencing (7–9). At present, the research on the molecular mechanism of GC is relatively abundant, but finding diagnostic markers remains an important challenge. Uncovering key gene molecules and targeted mechanisms can provide more ideas for diagnosis and treatment of GC (10–12).

The N-Myc downstream regulatory gene (NDRG) family played multiple roles in cell proliferation, differentiation, and hypoxia-induced cancer metabolism (13). NDRG4 gene is a member of the NDRG family (14). The expression of NDRG4 gene served a crucial function in regulating the proliferation and apoptosis of tumor cells (15). Abnormal expression of NDRG4 was associated with colorectal cancer, pancreatic cancer, esophageal adenocarcinoma, and breast cancer (15–19). Previous studies showed that NDRG4 methylation can serve as a potential biomarker for colorectal cancer (20, 21). Currently, only one study explored the relationship between NDRG4 gene methylation and GC using tumor tissues and adjacent normal tissues (22). Considering the convenience for the early diagnosis of GC, peripheral blood is an advantageous sample type (23). Regrettably, no studies explored the association between NDRG4 gene promoter methylation and GC risk in peripheral blood leukocytes (PBLs).

In this study, bioinformatics was used to analyze the roles of NDRG family genes in GC. Subsequently, two-phase case-control study was applied to explore the relation between methylation level of NDRG4 gene and GC risk. Meanwhile, the correlation of NDRG4 methylation with chemotherapy efficacy and prognosis was investigated. The expression of NDRG4 gene was compared between the hypermethylation and hypomethylation groups. Finally, the differences of methylation between rs7202037 genotypes were compared.

## 2 Methods

### 2.1 Bioinformatics analysis

#### 2.1.1 Bioinformatics analysis of NDRG family genes

The expression levels of NDRG family genes (NDRG1, NDRG2, NDRG3, NDRG4) were compared. The transcriptional expression data were obtained from the TCGA stomach adenocarcinoma (STAD) cohort, which included both non-paired samples (413 tumor tissues and 35 adjacent normal tissues) and paired samples (32 pairs, all of which were tumor tissues and adjacent normal tissues from the same patient). The data type was RNA-seq expression data, and the RSEM (RNA-seq by Expectation Maximization) quantification values after batch correction were used. To ensure the reliability of the analysis, all data were log_2_(x+1) transformed before analysis and standardized within the stomach adenocarcinoma cancer type using the z-score normalization formula (z = (x − μ)/σ, where x is the original quantification value, μ is the mean of the gene within this cancer type, and σ is the standard deviation of the gene within this cancer type). In this study, the median split method was used to group the patients in the TCGA-STAD cohort based on their expression levels.

To clarify the expression distribution characteristics of NDRG4 in different cell types within the tumor microenvironment of gastric tissue, the cell type-specific expression of GSE134520 was analyzed using the TISCH2 online tool (24). The raw count expression matrix was downloaded from the GEO database, and low-quality cells with total UMI counts < 1000 or detected gene numbers < 500 were excluded. Batch effects were evaluated using an entropy-based metric, and datasets with median entropy < 0.7 were corrected using Seurat v3.1.2. The top 2000 highly variable genes were selected and dimensionally reduced by principal component analysis (PCA). Cell clustering was performed using KNN and Louvain algorithms, and the results were visualized using the UMAP algorithm. Cell type annotation was conducted using the MAESTRO pipeline based on reference cell type characteristics and marker gene expression patterns. Differentially expressed genes in each cell cluster were identified using the Wilcoxon rank sum test. GSE134520 dataset contains 13 samples of gastric tissues at different pathological stages. GSCA online tool was used to visually analyze the expression trend of NDRG family genes in different pathological stages of GC.

#### 2.1.2 Immune-related signatures of NDRG family genes in GC

The easier package was used to calculate the scores of chemokines, cytotoxicity (CYT), interferons (INFγ), T-cell inflamed, and tertiary lymphoid structures (TLS). We compared the immune index scores between high and low expression groups of NDRG family genes. Groups were grouped according to the median expression of NDRG family genes. This study utilized the TIMER2.0 online platform for immune infiltration analysis (25). The core algorithm of TIMER was employed along with the CIBERSORT and EPIC algorithms for cross-validation. The input data was a TPM standardized gene expression matrix without logarithmic transformation, which is the officially designated standard input format for TIMER2.0. The raw expression data was extracted from the RSEM quantification results in the TCGA database. Spearman rank correlation analysis was conducted through the Gene Module module of the platform to quantify the association strength between NDRG4 expression and the infiltration level of immune cells.

#### 2.1.3 Methylation and expression of NDRG family genes in GC

Using TCGA methylation data and gene expression data, the methylation levels of multiple CpG sites in the promoter region of the NDRG family genes were analyzed (26). For NDRG family genes, the beta values of CpG sites in its promoter region were extracted from the TCGA methylation data, and the average beta value of all the displayed CpG sites was also calculated. Mean represented the mean value of methylation. To systematically assess the epigenetic-transcriptional axis in NDRG family genes, we quantified pairwise correlations between promoter DNA methylation β values and corresponding transcript abundances using Spearman’s rank correlation method.

#### 2.1.4 Survival analysis of NDRG family genes in GC

Clinical and transcriptomic data of GC were obtained from TCGA database. The GCSA online tool was used to analyze the survival differences between the high expression group and low expression group of NDRG family genes. Kaplan–Meier curves were generated with the R survival package to estimate survival probabilities. Kaplan-Meier survival analysis was used to compare disease-specific survival (DSS) and progression-free interval (PFI) between patients with high and low NDRG4 gene expression. Furthermore, we also evaluated the impact of different combinations of methylation and gene expression levels on DSS and PFI.

### 2.2 Correlation analysis of NDRG4 gene methylation in PBLs with the GC risk, chemotherapy efficacy, and prognosis

#### 2.2.1 Study subjects

In the primary screening phase, a total of 100 GC patients and 100 healthy controls (HCs) were enrolled. In the validation phase, enrolling 210 GC patients and 200 HCs. Finally, the study subjects enrolled in the two phases were combined for analysis. A total of 69 patients with advanced GC were selected, all of whom received chemotherapy with platinum plus fluoropyrimidine-based drugs (PPFs). We presented the design structure and sample distribution (**Figure 4A**). Meanwhile, we followed up 310 GC patients. The expression levels of NDRG4 gene in PBLs of 32 GC patients was detected. We genotyped the rs7202037 in 280 GC patients. All tumors were histologically diagnosed as adenocarcinoma with the stomach. Those with a pathological diagnosis of gastric stromal tumor, gastric lymphoma, or esophagogastric junction cancer were excluded. All patients had comprehensive clinicopathological data. Eligible patients had received no prior chemotherapy, radiotherapy, immunotherapy, or molecular targeted therapy before enrollment (27). All subjects voluntarily signed the informed consent form. This study was approved by the ethics committee of Anhui medical university (20160234). The estimation method of the sample size and the results were detailed in the **Supplementary Material** (28).

#### 2.2.2 Methylation detection

DNA was extracted from PBLs using FlexiGene DNA Kit (CatNo.51204, QIAGEN, Germany). Then, bisulfite conversion of DNA was performed using the EZ DNA Methylation-Gold Kit (CatNo.D5006, ZYMO Research, CA, USA) according to the protocol. The study utilized the EMBOSS Explorer software to predict CpG islands in the NDRG4 gene region. The screening criteria were as follows: (1) Observed/Expected ratio > 0.60. (2) C+G base percentage > 50%. (3) sequence length > 200 bp. (4) The analysis region was set to the key regulatory area from 2000 bp upstream of the gene transcription start site to 1000 bp downstream of the first exon.Following standard procedures (29). Based on these criteria, specific primer pairs were designed using Primer3.0 software (forward: 5’-GTTGGGATGGGGATGTTT-3’; reverse: 5’-CCCCRCCRACTTCTCACC-3’). Then, multiple PCR amplification and sequencing library construction were carried out using bisulfite-treated DNA as the template, successfully covering 17 CpG methylation sites within the target region. Finally, sequencing was performed on the Illumina HiSeq 2500 platform.

#### 2.2.3 Evaluation of chemotherapy efficacy

This study enrolled 69 GC patients to analyze the association between NDRG4 gene methylation and chemotherapy efficacy. The 69 subjects in this study met the following three inclusion criteria.

First, the GC patients had not received chemotherapy before enrollment. Second, they must be GC patients with stage IV. Third, PPFs was used as the first-line chemotherapy regimen after enrollment. Efficacy assessments were performed on patients every two chemotherapy cycles. Efficacy was evaluated in accordance with the RECIST1.1 (30). Finally, the assessment results were divided into a progressive disease group and a non-progressive disease group, among which the non-progressive group included GC patients with complete response, partial response, and stable disease.

#### 2.2.4 Evaluation of prognosis

In this study, 310 GC patients were followed up. Overall survival (OS) was used as the evaluation index to analyze the prognosis of all subjects. Disease-free survival (DFS) served as a proxy for postsurgical prognosis in stage I–III of GC. For GC patients with stage IV, progression-free survival (PFS) was used to evaluate their prognosis. Patients who underwent radical gastrectomy were followed up every two months during the first two postoperative years and every six months thereafter. Patients with stage IV received prognostic reassessment every two chemotherapy cycles. Patients were included starting from 2017, and the follow-up was conducted until March 31, 2025. OS, DFS, and PFS were measured in days. For DFS in stages I-III and OS, the multivariate Cox regression adjusted for age, gender, smoking, drinking, degree of differentiation, and TNM stage; for PFS in stage IV, it adjusted for age, gender, smoking, drinking, and degree of differentiation.

#### 2.2.5 Quantitative real-time PCR

The collected fresh blood samples were centrifuged, and peripheral blood mononuclear cells (PBMCs) were isolated from the blood samples using human peripheral blood lymphocyte separation medium (CatNo.P8610, Solarbio, Beijing, China). RNA was extracted from PBMCs, and its concentration and purity were measured using a NanoDrop 2000 spectrophotometer. Then, the target RNA was taken and reverse transcribed to synthesize cDNA using the StarScript Pro All-in-one RT Mix with gDNA Remover (CatNo.P401-02, Kangrun, Beijing, China). Finally, real-time quantitative PCR was performed using forward and reverse primers and the cDNA template. GAPDH was used as the internal reference gene, and all samples were subjected to three technical replicates. The cDNA of each sample was amplified in parallel by RT-PCR three times, and the Ct value of each reaction was recorded. During data analysis, the average of the Ct values of the three technical replicates was first calculated, and then the relative expression level of the target gene relative to GAPDH was calculated using the 2^(-ΔΔCt) method. Primer sequences, forward:5′-TAAGGGGCTCAGTCCTCCTC-3′, reverse:5′-GGAATGGCCTGGATCCGTAG-3′.

#### 2.2.6 Selection and detection of SNP

To investigate the effect of SNP on NDRG4 gene methylation, we focused on the tag SNP within the promoter regions of NDRG4 gene. The screening criteria were as follows: in the Chinese Han population, the minor allele frequency (MAF) was more than 5%, and the linkage disequilibrium coefficient (*r*²) was more than 0.8 (31). Eventually, the rs7202037 was selected. This locus is physically located in the upstream regulatory region of the NDRG4 gene (chr16:58,459,066-58,463,066), which is a key area for gene expression regulation. Public database analysis shows that rs7202037 is a significant expression quantitative trait locus (eQTL) and chromatin accessibility quantitative trait locus (caQTL) for NDRG4. This indicates that the genotype variation at this locus is not only significantly associated with the expression level of NDRG4 but also directly alters the openness of local chromatin, providing direct evidence for its regulatory function. This locus is within the binding motifs of multiple transcription factors, and its allelic variation can disrupt or enhance the binding ability of key transcription factors such as EZH2 and SUZ12, thereby affecting the transcriptional regulation of NDRG4. rs7202037 is located at position 58496970 on chromosome 16. Genotyping of the rs7202037 was performed by improved Multiplex Ligation-dependent Probe Amplification Reaction (iLMDR) technology (32). DNA was extracted from PBLs using a QIAGEN kit. Specific probes were designed to hybridize with the target SNP. The genotyping results were analyzed by capillary electrophoresis.

### 2.3 Statistical analysis

All statistical analyses were conducted using R (v4.3.3) and SPSS 26.0. For categorical variables, frequency (percentage) (n (%)) was used for description. For quantitative data, the Shapiro-Wilk test was first used to analyze whether the data met the normal distribution. Data meeting the normal condition were described using mean ± standard deviation (x ± s), and the two independent samples t-test or analysis of variance was used to compare the differences between two or more groups. Data not meeting the normal condition were described using median and upper and lower quartiles (M (P25, P75)). Logistic regression was employed to analyze the association between NDRG4 gene methylation level and GC risk. Cox regression was utilized to analyze the association between methylation and prognosis of GC patients. Benjamini-Hochberg (BH) correction was used to adjust the *P*-value and reduce the false discovery rate (FDR). The *χ*^2^ test was used to determine whether the frequency of SNP genotypes conformed to Hardy-Weinberg equilibrium (HWE). The Kruskal-Wallis test was employed to compare differences in the distribution of methylation levels among different SNP genotypes. In the study, *P* < 0.05 was considered significant.

## 3 Results

### 3.1 Bioinformatics analysis of NDRG family genes

#### 3.1.1 Expression of NDRG family genes in GC

**Figure 1:**
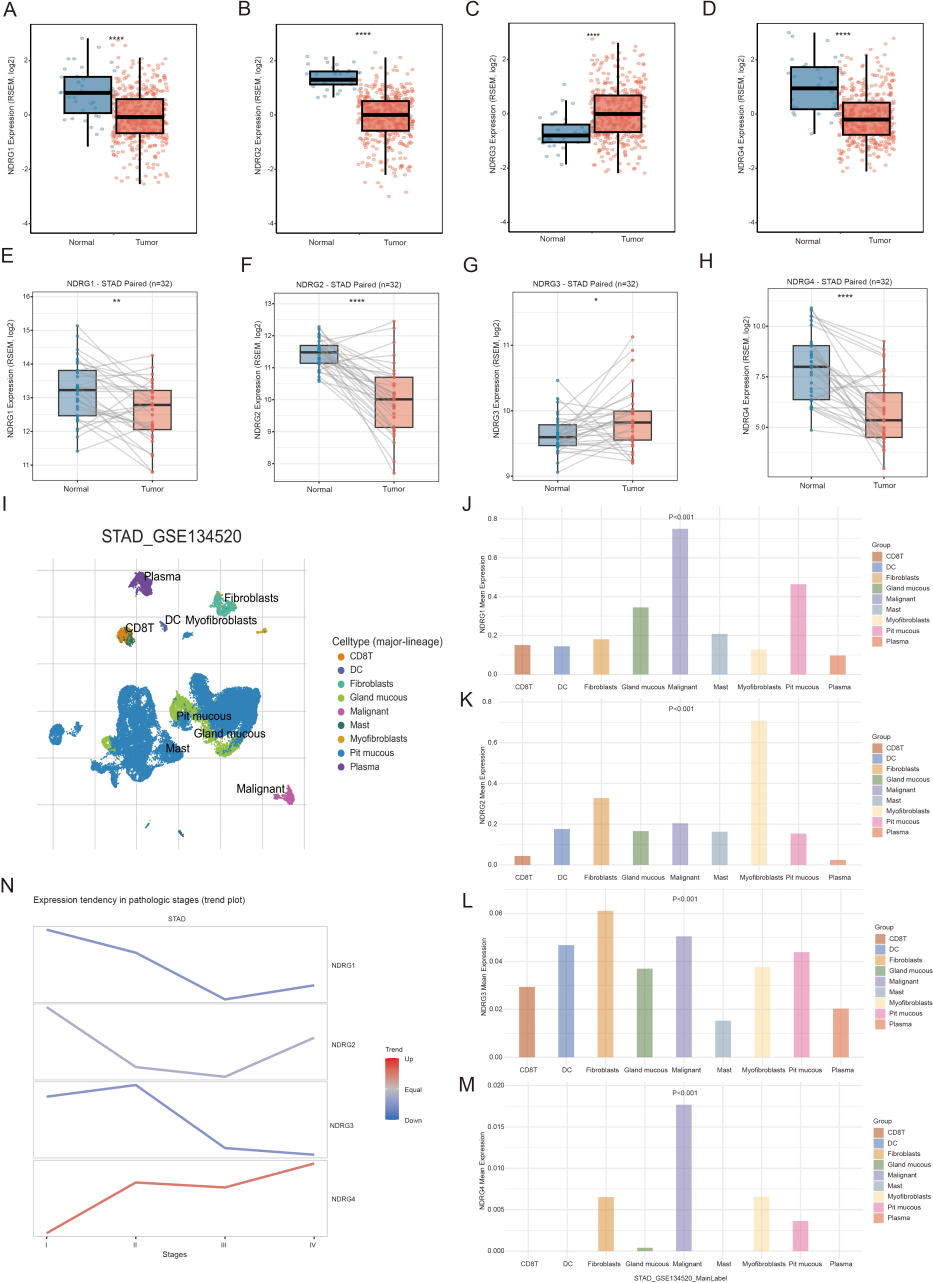
Expression of NDRG family genes in gastric cancer. **(A–D)** The expression levels of NDRG family genes in normal and gastric cancer tissues from TCGA dataset. **(E–H)** Expression connection diagrams of NDRG family genes in paired normal and gastric cancer tissues. **(I)** Single-cell clustering dimensionality reduction plot of gastric cancer dataset STAD_GSE134520. **(J–M)** NDRG family genes expression in different cell types of gastric cancer tissues. **(N)**Trend plot illustrating the expression tendency of NDRG family genes across I-IV stages of gastric cancer. *P < 0.05, **P < 0.01, ***P < 0.001, ****P < 0.0001.

The results showed that NDRG1, NDRG2 and NDRG4 were highly expressed in normal tissues, while NDRG3 exhibited high expression in tumor tissues (*P* < 0.05) (**Figures 1A–D**). Subsequently, the consistency of this differential expression was further verified using paired samples (*P* < 0.05) (**Figures 1E–H**). Single-cell specific expression analysis revealed that GC tumor tissues were mainly composed of pit mucous cells, fibroblasts, and malignant cells (**Figure 1I**). NDRG family genes are highly expressed in malignant cells, fibroblasts, and cancer-associated fibroblasts (CAFs) in GC tissues. (*P* < 0.001) (**Figures 1J–M**). The expression trend of NDRG family genes in different pathological stages of GC was compared. The results showed that the expression of NDRG4 increased with the progression of pathological stage of GC (**Figure 1N**).

#### 3.1.2 Immune-related signatures of NDRG family genes in GC

**Figure 2:**
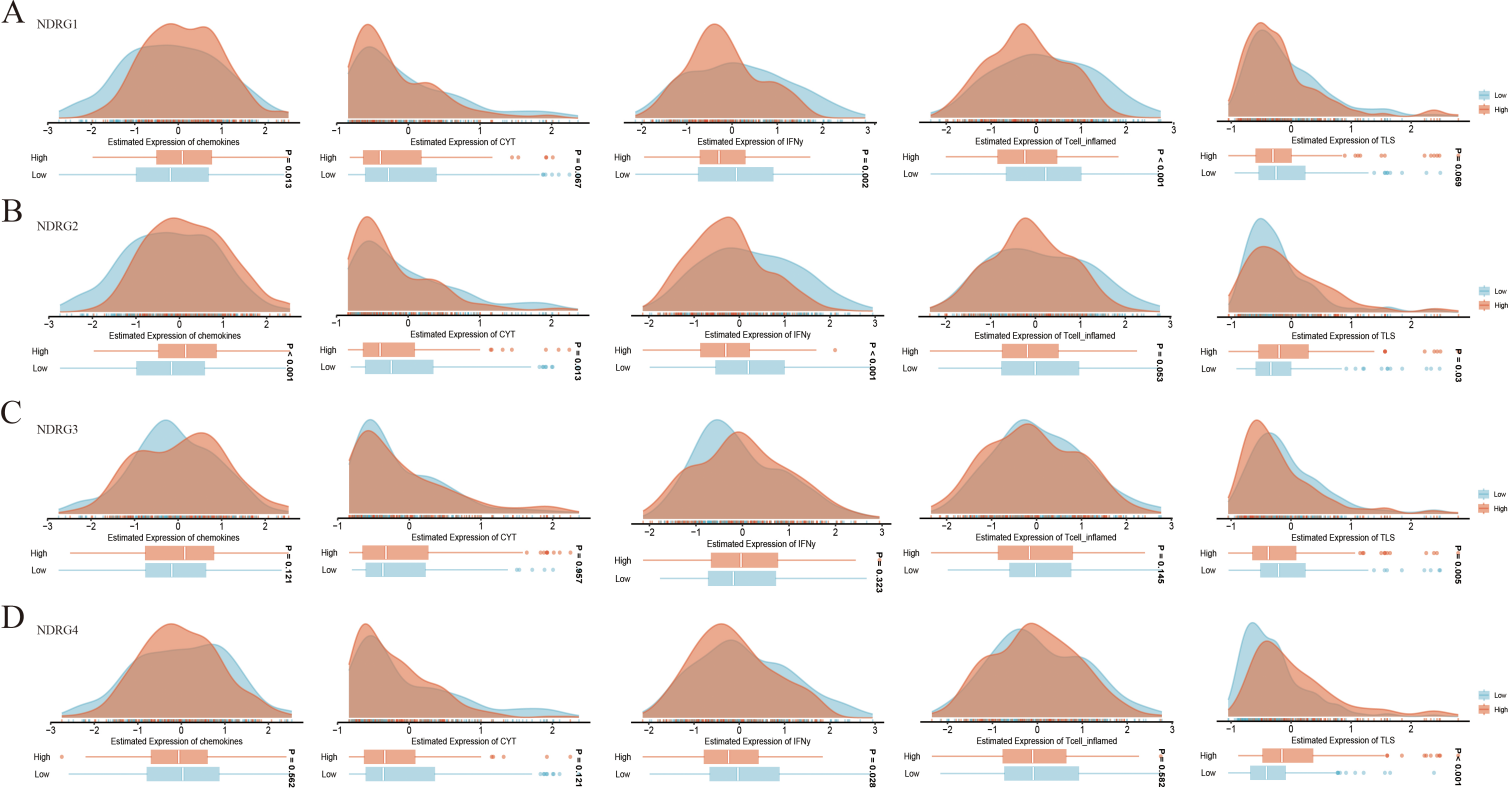
Immune-related signatures of NDRG family genes in gastric cancer. **(A)** NDRG1 gene. **(B)** NDRG2 gene. **(C)** NDRG3 gene. **(D)** NDRG4 gene. Density and box plots showing the estimated scores of chemokines, cytotoxicity (CYT), interferon-γ (IFNγ), T cell_inflamed, and tertiary lymphoid structures (TLS) in NDRG family genes low and high expression groups. “Low” and “High” represent NDRG family genes expression levels, with *P* values indicating statistical significance.

The expression levels of NDRG family genes were correlated with immune-related signatures of GC. For the NDRG1 gene, the chemokine score was higher in the high expression group, while the scores of INFγ and the T cell-inflamed were higher in the low expression group (*P* < 0.05) (**Figure 2A**). For the NDRG2 gene, the scores of chemokines and TLS were higher in the high expression group, while the scores of INFγ and CYT were higher in the low expression group (*P* < 0.05) (**Figure 2B**). For the NDRG3 gene, the score of TLS (*P* < 0.05) was higher in the low expression group (**Figure 2C**). For the NDRG4 gene, the score of TLS (*P* < 0.05) was higher in the high expression group, while the score of INFγ (*P* < 0.05) was higher in the low expression group (**Figure 2D**). This graph showed the association of NDRG4 gene with various immune cell types (**Supplementary Figure S1A**). The expression level of NDRG4 gene was positively correlated with the infiltration of CAFs, endothelial and CD4+ T cells, but negatively correlated with CD4+ Th2 cells (*P* < 0.05) (**Supplementary Figures S1B–I**).

#### 3.1.3 Methylation of NDRG family genes in GC

**Figure 3:**
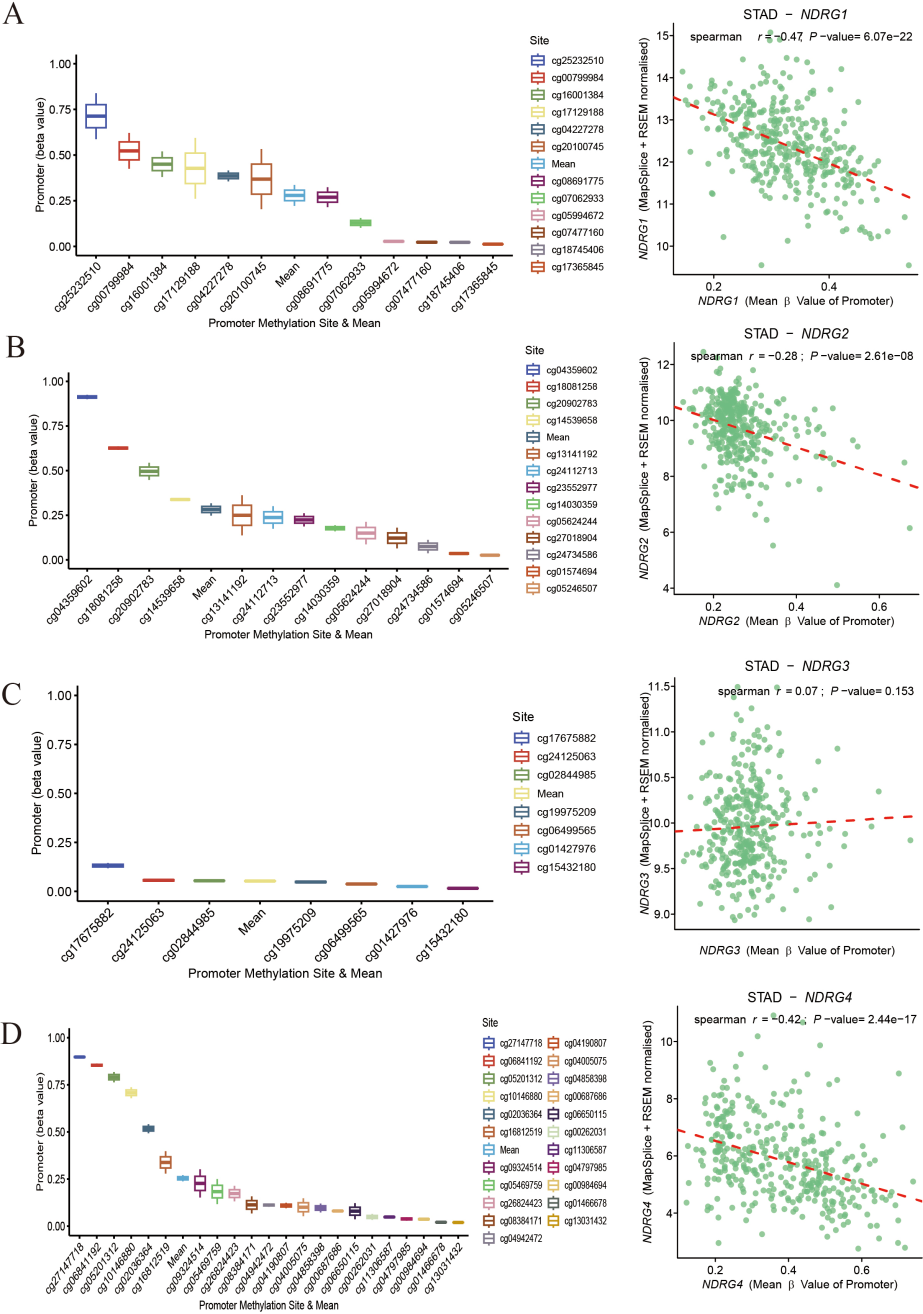
Methylation and expression of NDRG family gene in gastric cancer. **(A)** NDRG1 gene. **(B)** NDRG2 gene. **(C)** NDRG3 gene. **(D)** NDRG4 gene. In each part, the left showing the distribution of different promoter methylation sites and the average methylation level (measured by beta value) of the corresponding gene, with different colors representing different methylation sites; the right side presenting the correlation between gene expression and the average methylation level of the promoter. The red dashed line is the fitted trend line, and the marked Spearman correlation coefficient (*r*) and *P*-value reflect the strength and significance of the correlation.

The methylation levels of different CpG sites were different. For NDRG1, NDRG2 and NDRG4 genes, significant differences in methylation levels were observed across multiple CpG sites. Interestingly, the average methylation levels of the CpG sites of the NDRG1 (*r* = -0.47, *P* = 6.07×10^-22^), NDRG2 (*r* = -0.28, *P* = 2.61×10^-8^), and NDRG4 (*r* = -0.42, *P* = 2.44×10^-17^) genes were negatively correlated with its expression levels (**Figures 3A, B, D**). For the NDRG3 gene, the methylation levels at each CpG sites were low, and there was no correlation between its methylation and the gene expression (*r* = 0.07, *P* = 0.153) (**Figure 3C**).

#### 3.1.4 Prognosis of NDRG family genes in GC

In this study, survival differences between different expression groups of NDRG family genes were compared. Among the NDRG family genes, only NDRG4 showed a significant association with DSS and PFI (**Supplementary Figure S2A**). The Kaplan-Meier curves showed that patients with high expression of NDRG4 gene had poorer DSS and PFI compared with low expression (*P* < 0.05) (**Supplementary Figures S2B, C**). Furthermore, the survival prognosis of GC patients in the high methylation and low expression group was superior to that of patients in the low methylation and high expression group. (*P* < 0.05) (**Supplementary Figures S2D, E**).

### 3.2 Correlation analysis of NDRG4 gene methylation in PBLs with the GC risk, chemotherapy efficacy, and prognosis

#### 3.2.1 Methylation of NDRG4 gene and GC risk in PBLs

##### 3.2.1.1 Primary screening phase

Methylation sequencing results showed that there were 17 methylation sites in NDRG4 gene (**Supplementary Table S1**). **Table 1** and **Supplementary Table S2** presented the information of all the subjects. Statistical tests showed that there were no significant differences in age and gender between the case group and the control group in the two-phase study (**Table 1**). Primary screening phase showed that the methylation levels of nine sites (NDRG4-chr16:58497230: *OR* = 1.425, 95%*CI* = 1.091-1.862, *P* = 0.009; NDRG4-chr16:58497236: *OR* = 2.314, 95%*CI* = 1.388-3.855, *P* = 0.001; NDRG4-chr16:58497239: *OR* = 4.521, 95%*CI* = 2.065-9.897, *P* = 1.61×10^-4^; NDRG4-chr16:58497251: *OR* = 2.697, 95%*CI* = 1.149-6.331, *P* = 0.023; NDRG4-chr16:58497262: *OR* = 2.034, 95%*CI* = 1.106-3.742, *P* = 0.022; NDRG4-chr16:58497265: *OR* = 2.188, 95%*CI* = 1.268-3.774, *P* = 0.005; NDRG4-chr16:58497325: *OR* = 1.474, 95%*CI* = 1.113-1.951, *P* = 0.007; NDRG4-chr16:58497329: *OR* = 1.626, 95%*CI* = 1.111-2.381, *P* = 0.012; NDRG4-chr16:58497332:*OR* = 1.392, 95%*CI* = 1.088-1.781, *P* = 0.008) were associated with GC risk (**Table 2**). Meanwhile, the average (NDRG4-gene: *OR* = 4.297, 95%*CI* = 1.955-9.444, *P* = 2.85×10^-4^) methylation levels of the 17 CpG sites was also associated with GC risk (**Table 2**).

**Table 1:** Age and gender information of gastric cancer patients and healthy controls.

Characteristic	Preliminary screening stage			Validation stage			Combination (preliminary+validation)		
	GC patients (n=100)	HCs (n=100)	*P-*value	GC patients (n=210)	HCs (n=200)	*P-*value	GC patients (n=310)	HCs (n=300)	*P-*value
Age (Year, Mean±SD)	61.25 ± 11.36	60.84 ± 11.63	0.523	60.64 ± 10.42	60.39 ± 10.32	0.654	60.84 ± 10.71	60.54 ± 10.76	0.731
Gender, n (%)									
Male	70 (70.00)	70 (70.00)	1.000	150 (71.43)	144 (72.00)	0.896	220 (70.97)	214 (71.33)	0.920
Female	30 (30.00)	30 (30.00)		60 (28.57)	56 (28.00)		90 (29.03)	86 (28.67)	

**Table 2:** Association between NDRG4 gene/sites methylation and gastric cancer risk in primary screening phase.

Gene/sites	Methylation level^a^		Logistic regression analysis			
	GC (n=100)	HCs (n=100)	Crude *OR * (95%*CI*)	Crude *P-*value	Adjusted *OR* (95%*CI*)^*^	Adjusted *P*-value^*^
NDRG4-gene	1.69 (1.49-2.03)	1.54 (1.28-1.75)	3.944 (1.855-8.387)	3.64×10^-4^	4.297 (1.955-9.444)	**2.85×10^-4^**
NDRG4-chr16:58497230	2.32 (1.77-3.30)	1.98 (1.44-2.62)	1.412 (1.087-1.836)	0.010	1.425 (1.091-1.862)	**0.009**
NDRG4-chr16:58497236	1.65 (1.24-2.07)	1.45 (1.08-1.77)	2.316 (1.389-3.861)	0.001	2.314 (1.388-3.855)	**0.001**
NDRG4-chr16:58497239	0.99 (0.78-1.22)	0.78 (0.49-1.03)	4.145 (1.945-8.835)	2.31×10^-4^	4.521 (2.065-9.897)	**1.61×10^-4^**
NDRG4-chr16:58497251	0.61 (0.45-0.82)	0.51 (0.38-0.66)	2.653 (1.145-6.144)	0.023	2.697 (1.149-6.331)	**0.023**
NDRG4-chr16:58497259	1.05 (0.80-1.31)	0.89 (0.67-1.12)	1.768 (1.000-3.127)	0.050	1.767 (0.996-3.136)	0.052
NDRG4-chr16:58497262	1.15 (0.85-1.47)	0.92 (0.65-1.14)	2.028 (1.108-3.712)	0.022	2.034 (1.106-3.742)	**0.022**
NDRG4-chr16:58497265	1.37 (1.07-1.69)	1.16 (0.94-1.45)	2.172 (1.266-3.729)	0.005	2.188 (1.268-3.774)	**0.005**
NDRG4-chr16:58497267	0.92 (0.74-1.14)	0.85 (0.62-1.10)	1.397 (0.763-2.557)	0.278	1.395 (0.760-2.560)	0.282
NDRG4-chr16:58497269	1.04 (0.70-1.42)	0.94 (0.69-1.26)	1.394 (0.867-2.241)	0.171	1.406 (0.860-2.299)	0.174
NDRG4-chr16:58497292	1.59 (1.18-2.08)	1.50 (1.26-1.89)	1.210 (0.833-1.758)	0.318	1.207 (0.829-1.757)	0.327
NDRG4-chr16:58497304	1.81 (1.40-2.19)	1.63 (1.30-1.93)	1.487 (0.975-2.269)	0.066	1.508 (0.974-2.335)	0.066
NDRG4-chr16:58497309	2.00 (1.56-2.76)	1.80 (1.37-2.34)	1.272 (0.945-1.711)	0.112	1.283 (0.942-1.748)	0.114
NDRG4-chr16:58497325	3.07 (2.65-3.58)	2.74 (2.20-3.27)	1.459 (1.108-1.921)	0.007	1.474 (1.113-1.951)	**0.007**
NDRG4-chr16:58497327	1.51 (1.16-1.98)	1.51 (1.18-1.84)	1.242 (0.828-1.863)	0.296	1.243 (0.817-1.891)	0.310
NDRG4-chr16:58497329	1.94 (1.47-2.52)	1.69 (1.29-2.20)	1.619 (1.111-2.360)	0.012	1.626 (1.111-2.381)	**0.012**
NDRG4-chr16:58497332	3.70 (3.21-4.75)	3.57 (2.74-4.09)	1.389 (1.088-1.774)	0.008	1.392 (1.088-1.781)	**0.008**
NDRG4-chr16:58497337	1.71 (1.27-2.29)	1.61 (1.18-2.02)	1.067 (0.791-1.439)	0.673	1.063 (0.782-1.443)	0.698

a Methylation level is expressed as a percentage, data was expressed as median (*P*_25_, *P*_75_). ^*^Adjusted for age and sex. GC, gastric cancer; HCs, health controls; CI, confidence interval; OR, odds ratio.

The bolded values are to highlight that the P value is less than 0.05.

##### 3.2.1.2 Validation phase

Based on the nine correlated sites in primary screening phase, this study further conducted validation phase analysis. Validation phase showed that the methylation levels of four sites (NDRG4-chr16:58497239: *OR* = 2.536, 95%*CI* = 1.514-4.248, *P* = 4.07×10^-4^; NDRG4-chr16:58497251: *OR* = 1.883, 95%*CI* = 1.053-3.370, *P* = 0.033; NDRG4-chr16:58497262: *OR* = 1.910, 95%*CI* = 1.223-2.981, *P* = 0.004; NDRG4-chr16:58497325:*OR* = 1.217, 95%*CI* = 1.012-1.464, *P* = 0.036) were associated with GC risk (**Table 3**). Meanwhile, the average (NDRG4-gene: *OR* = 1.893, 95%*CI* = 1.075-3.334, *P* = 0.027) methylation levels of the 17 CpG sites was also associated with GC risk (**Table 3**). After adjustment by BH method, there were still 2 sites (NDRG4-chr16:58497239: *P*_BH_=0.004; NDRG4-chr16:58497262, *P*_BH_=0.02) whose methylation levels were associated with GC risk (**Table 3**).

**Table 3:** Association between NDRG4 gene/sites methylation and gastric cancer risk in validation phase.

Gene/sites	Methylation level^a^		Logistic regression analysis				*P*_BH_
	GC (n=210)	HCs (n=200)	Crude *OR (*95%*CI)*	Crude *P-*value	Adjusted *OR* (95%*CI*)^*^	Adjusted *P*-value^*^	
NDRG4-gene	1.58 (1.38-1.86)	1.52 (1.35-1.77)	1.849 (1.068-3.202)	0.028	1.893 (1.075-3.334)	**0.027**	0.072
NDRG4-chr16:58497230	1.98 (1.61-2.70)	2.01 (1.55-2.67)	1.076 (0.884-1.308)	0.465	1.073 (0.881-1.308)	0.484	0.605
NDRG4-chr16:58497236	1.50 (1.12-1.96)	1.42 (1.08-1.73)	1.195 (0.913-1.564)	0.195	1.194 (0.910-1.566)	0.200	0.286
NDRG4-chr16:58497239	0.89 (0.71-1.12)	0.85 (0.61-1.01)	2.541 (1.517-4.256)	3.97×10^-4^	2.536 (1.514-4.248)	**4.07×10^-4^**	**0.004**
NDRG4-chr16:58497251	0.57 (0.44-0.84)	0.54 (0.40-0.70)	1.862 (1.047-3.309)	0.034	1.883 (1.053-3.370)	**0.033**	0.072
NDRG4-chr16:58497262	1.01 (0.76-1.29)	0.93 (0.70-1.12)	1.907 (1.223-2.974)	0.004	1.910 (1.223-2.981)	**0.004**	**0.020**
NDRG4-chr16:58497265	1.26 (1.01-1.55)	1.22 (0.91-1.46)	1.422 (0.956-2.116)	0.082	1.423 (0.952-2.127)	0.085	0.142
NDRG4-chr16:58497325	3.02 (2.48-3.66)	2.83 (2.34-3.47)	1.216 (1.015-1.458)	0.034	1.217 (1.012-1.464)	**0.036**	0.072
NDRG4-chr16:58497329	1.81 (1.35-2.28)	1.77 (1.41-2.22)	0.951 (0.773-1.171)	0.638	0.947 (0.767-1.170)	0.615	0.683
NDRG4-chr16:58497332	3.58 (3.03-4.38)	3.59 (2.95-4.22)	1.016 (0.893-1.155)	0.812	1.014 (0.890-1.155)	0.833	0.833

a Methylation level is expressed as a percentage, data was expressed as median (*P*_25_, *P*_75_). ^*^Adjusted for age and sex. GC, gastric cancer; HCs, health controls; CI, confidence interval; OR, odds ratio; BH, Benjamini-Hochberg.

The bolded values are to highlight that the P value is less than 0.05.

##### 3.2.1.3 Combined analysis

The combined analysis demonstrated that the methylation levels of NDRG4-chr16:58497239 (*OR* = 3.016, 95%*CI* = 1.967-4.625, *P*_BH_=8.38×10^-7^) and NDRG4-chr16:58497262 (*OR* = 1.939, 95%*CI =*1.356-2.774, *P*_BH_=2.87×10^-4^) associated with GC risk (**Table 4**). A nomogram model was constructed to predict GC risk (**Supplementary Figure 3A**). The AUC value of the nomogram was 0.647 (95%*CI*: 0.604-0.690) (**Supplementary Figure S3B**). The calibration curve was close to the ideal line (**Supplementary Figure S3C**). The standardized net benefit of the nomogram model was greater than the strategies of all patients intervene or all patients do not intervene (**Supplementary Figure 3D**). In addition, the methylation level of NDRG4 gene in PBLs was not associated with the degree of differentiation, TNM stage, tumor location, CEA level or CA199 level of GC (*P*_BH_>0.05) (**Supplementary Tables S3**–**S7**).

**Table 4:** Combined analysis of the association between NDRG4 sites methylation and gastric cancer risk.

Sites	Methylation level^a^		Logistic regression analysis				*P*_BH_
	GC (n=310)	HCs (n=300)	Crude *OR (*95%*CI)*	Crude *P-*value	Adjusted *OR* (95%*CI*)^*^	Adjusted *P*-value^*^	
NDRG4-chr16:58497239	0.91 (0.73-1.17)	0.81 (0.57-1.01)	2.994 (1.955-4.586)	4.57×10^-7^	3.016 (1.967-4.625)	**4.19×10^-7^**	**8.38×10^-7^**
NDRG4-chr16:58497262	1.05 (0.80-1.36)	0.93 (0.68-1.13)	1.935 (1.355-2.764)	2.83×10^-4^	1.939 (1.356-2.774)	**2.87×10^-4^**	**2.87×10^-4^**

a Methylation level is expressed as a percentage, data was expressed as median (*P*_25_, *P*_75_). ^*^Adjusted for age and sex. GC, gastric cancer; HCs, health controls; CI, confidence interval; OR, odds ratio; BH, Benjamini-Hochberg.

The bolded values are to highlight that the P value is less than 0.05.

#### 3.2.2 Methylation level of NDRG4 gene and therapeutic efficacy of GC patients

**Figure 4:**
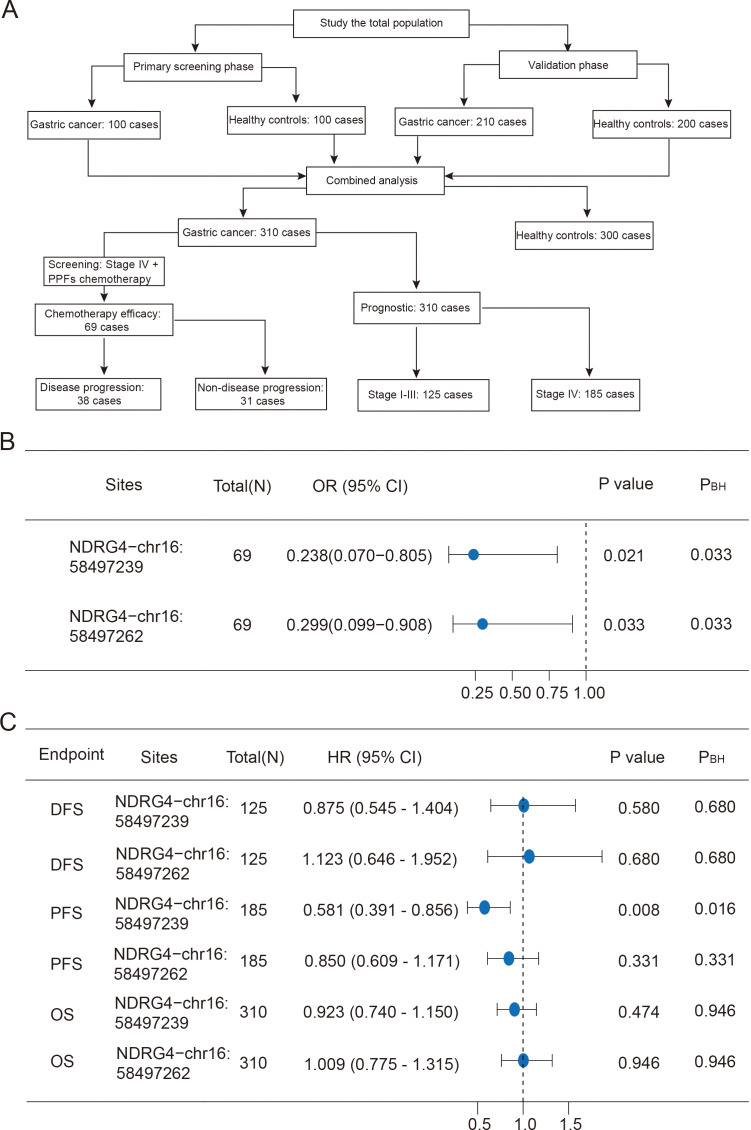
The association between methylation of the NDRG4 locus in peripheral blood leukocytes and the efficacy and prognosis of chemotherapy for gastric cancer. **(A)** Flowchart of research design and sample distribution. **(B)** Methylation of the NDRG4 sites and the efficacy of chemotherapy for gastric cancer. **(C)** Methylation of the NDRG4 sites and prognosis of gastric cancer. DFS, Disease-free survival; PFS, Progression-free survival; OS, Overall survival*. P*_BH_ denotes the Benjamini–Hochberg corrected *P*-value.

A total of 69 patients undergoing chemotherapy were included in this study. All patients had distant metastasis, and the median duration of treatment was 17 months. The study showed that the methylation levels of NDRG4-chr16:58497239 (*OR* = 0.238, 95%*CI* = 0.070-0.805, *P* = 0.021) and NDRG4-chr16:58497262 (*OR* = 0.299, 95%*CI* = 0.099-0.908, *P* = 0.033) in GC were associated with chemotherapeutic efficacy. After BH correction, the result was remained statistically significant (*P*_BH_<0.05) (**Figure 4B**; **Supplementary Table S8**). This study constructed a nomogram prediction model for chemotherapeutic efficacy. The AUC value of the nomogram was 0.665 (95%*CI*: 0.536-0.793) (**Supplementary Figure S4**).

#### 3.2.3 Methylation level of NDRG4 gene and prognosis of GC patients

Among the 310 GC patients, 242 patients died at the end of follow-up. A total of 125 stage I-III patients who received surgical treatment, 81 patients recurred. Among 185 stage IV patients, 169 patients metastasized. For stage I-III patients, the median follow-up time for DFS was 861 (95%*CI* = 618-1104) days. For stage IV patients, the median follow-up time for PFS was 188 (95%*CI* = 147-229) days. The median follow-up time for OS of all patients was 858 (95%*CI* = 754-962) days. Cox regression analysis results showed that the methylation level of the NDRG4-chr16: 58497239 (*HR* = 0.577, 95%*CI* = 0.388-0.858, *P* = 0.008) was associated with PFS (**Supplementary Table S9**). After BH correction, it still showed a correlation (*P*_BH_=0.016) (**Figure 4C**). For DFS and OS, neither NDRG4-chr16:58497239 nor NDRG4-chr16:58497262 showed association with survival (*P*>0.05). To predict the individualized 3-year survival probability, a predictive model was constructed in this study. The AUC value of this model was 0.764 (95%*CI*: 0.616–0.912) (**Supplementary Figure S5**).

#### 3.2.4 Expression of NDRG4 gene in PBLs

**Figure 5:**
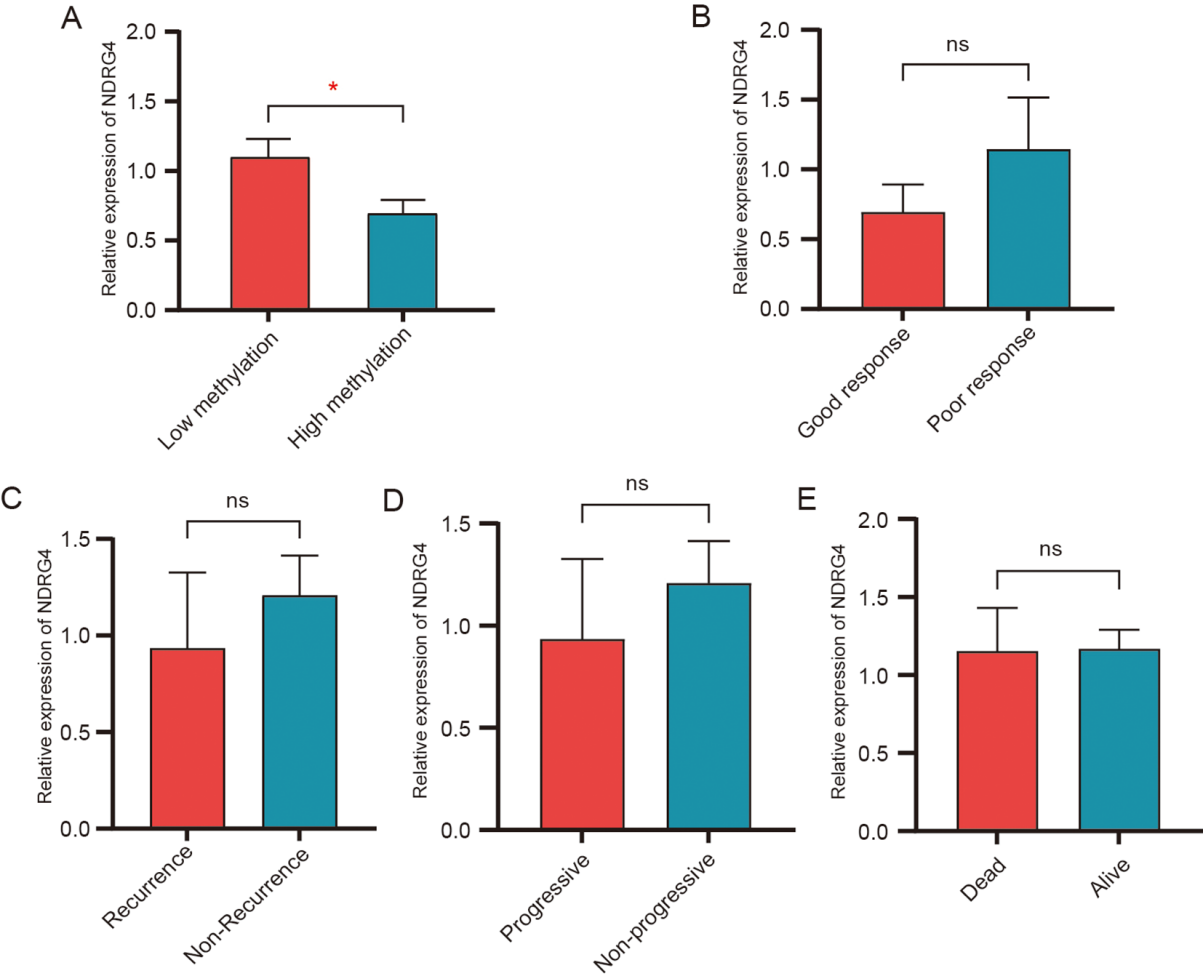
Association of NDRG4 gene expression with methylation and clinical outcomes. **(A)** Relative expression of NDRG4 in the low-methylation group and high-methylation group. **(B)** Relative expression of NDRG4 in patients with good response versus poor response to treatment. **(C)** Relative expression of NDRG4 in patients with recurrence versus non-recurrence. **(D)** Relative expression of NDRG4 in patients with progressive disease versus non-progressive disease. **(E)** Relative expression of NDRG4 in dead patients versus alive patients. *P < 0.05. ns, not significant.

In this study, peripheral blood samples of 32 GC patients were collected. The methylation level and expression level of NDRG4 gene were detected. Compared with the hypomethylation group, the relative expression of NDRG4 gene in the hypermethylation group was lower (*P* = 0.019) (**Figure 5A**). The follow-up results showed that among the 8 stage IV GC patients who had not undergone surgery and met the chemotherapy efficacy evaluation criteria, 4 had good efficacy and 4 had poor efficacy. There was no statistically significant difference in the expression level of NDRG4 between the two groups (*P*>0.05) (**Figure 5B**). Among the 14 stage I-III GC patients, 11 did not have recurrence and 3 had recurrence. There was no statistically significant difference in the expression level of NDRG4 between the recurrence group and the non-recurrence group (*P*>0.05) (**Figure 5C**). Among the 18 stage IV GC patients, 3 had no disease progression and 15 had disease progression. There was no statistically significant difference in the expression level of NDRG4 between the progression group and the non-progression group (*P*>0.05) (**Figure 5D**). Among all 32 patients, 24 survived and 8 died of GC. There was no statistically significant difference in the expression level of NDRG4 between the survival group and the death group (*P*>0.05) (**Figure 5E**). The expression level of NDRG4 in PBLs was not correlated with the chemotherapy efficacy, disease recurrence, disease progression or short-term survival status of patients with GC.

#### 3.2.5 Methylation level of NDRG4 gene and SNP genotypes

A total of 280 GC patients were genotyped for rs7202037, including 154 cases of AA genotype, 113 cases of AC genotype and 13 cases of CC genotype. The genotype frequency distribution conformed to the HWE test (*P* = 0.173). Based on the three genotypes, dominant (AA vs. AC+CC), recessive (AA+AC vs. CC), and additive (AA/AC/CC) models were constructed. Under the dominant model, the methylation levels of NDRG4-chr16:58497325 (*P*_BH_=0.028) showed a significant difference among genotypes. In the recessive model, NDRG4-chr16:58497239 (*P*_BH_=0.024) and NDRG4-chr16:58497325 (*P*_BH_=0.011) exhibited significant difference among genotypes. In the additive model, the methylation levels of NDRG4-chr16:58497239 (*P*_BH_=0.044) and NDRG4-chr16:58497325 (*P*_BH_=0.011) were different among the three genotypes (**Supplementary Table S10**).

## 4 Discussion

In the study, bioinformatics method was used to explore the function of NDRG family genes in GC. Given the significant role of NDRG4 gene, the study further investigated the association between methylation of NDRG4 gene in PBLs and GC risk, chemotherapy efficacy, and prognosis. Bioinformatics results showed that the expression levels of NDRG family genes in GC tissues and normal tissues were different. The expression of NDRG family genes was localized in GC malignant cells, fibroblasts and CAFs. NDRG family genes could affect the immune-related characteristics of GC. In addition, case-control and follow-up studies found that the methylation of the NDRG4 gene in PBLs was associated with the GC risk, chemotherapy efficacy, and prognosis. In peripheral blood mononuclear cells, the expression level of NDRG4 gene in the hypermethylated group was lower than that in the hypomethylated group. The methylation level of NDRG4 gene was different among different SNP (rs7202037) genotypes.

In recent years, the attention of NDRG family genes in the cancer field increased significantly. NDRG family genes were associated with liver cancer and breast cancer (14, 33). The study showed that the expression levels of NDRG family genes were different in GC tissues and normal tissues. The expression levels of NDRG1, NDRG2, and NDRG4 genes were significantly decreased in GC tissues, suggesting they may act as tumor suppressor genes for GC. The expression level of NDRG4 gene in GC was closely related to the process of tumor invasion and metastasis (15). The downregulation of NDRG4 gene expression weakened its tumor suppressor effect. Single-cell analysis localized the expression of NDRG4 gene in malignant cells, fibroblasts, and CAFs. Normal gastric epithelial cells can transform into malignant cells, and normal fibroblasts can transform into CAFs (34, 35). CAFs are the main components of the tumor microenvironment (TME). They can promote tumor growth and provide microenvironment support by secreting cytokines and reshaping the matrix structure (36, 37). Thus, the decreased expression of NDRG4 gene not only directly drives the malignant transformation of gastric mucosal epithelial cells, but also indirectly promotes the progression of GC by regulating the formation of the TME.

Immune-related signature analysis showed that the score of TLS was positively correlated with the expression level of NDRG4 gene. TLS are ectopic lymphoid aggregates, which are crucial for the prognosis and immunotherapy of cancer patients (38). Previous study showed that TLS can enhance the efficacy of tumor immunotherapy (39). In the TME of GC, total CD4^+^T cells are positively correlated with the expression level of NDRG4 gene. The result showed that NDRG4 gene can increase TLS by promoting the aggregation of CD4^+^T cells, which provided a new basis for understanding the formation mechanism of TLS and optimizing immunotherapy strategies.

The hypermethylation of NDRG4 gene can significantly downregulate its expression. Methylation specifically referred to the modification process of adding a methyl group to a cytosine (40). Methylation can affect the transcription initiation process of colorectal cancer related genes, and then change the expression level of these genes. This epigenetic regulatory mechanism is considered one of the factors driving the development of colorectal cancer (41). The development of GC may also be affected by this mechanism. DNA methyltransferase inhibitor, 5-azacytidine can reduce the aberrant hypermethylation in gene promoter regions, thereby effectively restoring and increasing the expression levels of relevant genes (42, 43). Based on the concept of translational medicine, the association between NDRG4 methylation and its expression identified in this study not only verifies its epigenetic regulatory characteristics, but also provided a potential intervention direction with both specificity and feasibility for the epigenetic treatment of GC.

Two-phase case-control study revealed that the hypermethylation of NDRG4-chr16:58497239 and NDRG4-chr16:58497262 in PBLs was associated with high risk of GC. This result was consistent with previous study on GC tissues, which showed that hypermethylation of the NDRG4 gene was high risk factor of GC (22). Compared with GC tissues, peripheral blood is not only easier to obtain but also non-invasive, making it more suitable for large sample collection. Meanwhile, studies showed that the methylation of specific genes in PBLs can serve as a non-invasive tumor biomarker (44, 45). Notably, the study showed that hypermethylation of NDRG4 gene in PBLs could downregulate its expression. This is consistent with the methylation and expression relationship observed in GC tissues (15, 22). This consistency indicated that NDRG4 gene methylation derived from peripheral blood can serve as a surrogate indicator for tissue level epigenetic changes, thereby overcoming the limitations of invasive tissue sampling. This study established a risk prediction model based on the methylation level of the NDRG4 gene, but its clinical application value is limited. At present, it can only be used as an auxiliary reference indicator. In the future, multiple institutions, independent external cohort validation is needed, and functional experiments should be combined to clarify its molecular mechanism, so as to further clarify its clinical application prospects.

This study found that high methylation is a protective factor for the therapeutic effect and prognosis in GC. NFAT5 K668 methylation in glioblastoma multiforme prevented degradation of NFAT5 and upregulated its target gene MGMT, which led to a poor response to temozolomide (TMZ) (46). Methylation could affect the efficacy of TMZ chemotherapy by regulating the expression of SNHG12 (47). These results suggested that methylation could affect drug efficacy in multiple ways, involving the regulation of gene expression or protein function related to drug metabolism or target response. The study showed that NDRG4 gene hypermethylation may enhance the efficacy of PPFs. DNA methylation is highly correlated with platinum drug resistance (48). Platinum drug bound to nuclear DNA and induced the death of malignant cells (49). Methylation of NDRG4 gene may influence drug efficacy through interfering with the binding between platinum and DNA. The specific mechanism still needs to be further explored.

SNP can affect gene methylation by altering the accessibility of CpG sites and the binding capacity of transcription factors (50, 51). rs7202037 is located in the upstream regulatory region of NDRG4 and has a high regulatory potential. It can regulate the function of NDRG4 through eQTL, chromatin openness and transcription factor binding. The study identified rs7202037, finding that the methylation level of NDRG4 gene was different among different genotypes. CC genotype at rs7202037 has higher methylation level than AA and AC genotypes. This may be attributed to the fact that cytosine residues were direct targets of DNA methyltransferases (52, 53). Previous study shown that SNP rs188303909 can regulate DNA methylation to regulate the expression of osteoporosis susceptibility gene EN1 (54). This suggested that SNP may affect the normal function of genes by regulating the methylation process. SNP in human genes can serve as risk factors for cancer development by affecting gene function and epigenetic modifications (55). Although the exact mechanism still needed to be explored in depth, this finding suggested that genetic variation could regulate the epigenetic state of NDRG4 gene.

The study had several strengths. First, the study was the first to explore the association between methylation of the NDRG4 gene in PBLs and the risk of GC, the efficacy of chemotherapy, and prognosis. Second, two-phase case-control study was conducted to explore the relationship between methylation of the NDRG4 gene in PBLs and the risk of GC. Third, this study was the first to investigate the effect of SNP rs7202037 on NDRG4 gene methylation in GC. This study has certain limitations. First, all members of the case and control study were basically from the same hospital. We will further validate the relevant conclusions through multiple institutions. Second, the sample size included in chemotherapy efficacy study may be insufficient. Third, as we did not collect information on the patients’ histological subtypes (intestinal type, diffuse type, and mixed type), we did not perform relevant analyses. Fourth, the causal relationship between methylation of NDRG4 gene and GC remained unclear. Fifth, the mechanism of methylation of NDRG4 gene in GC needed further exploration.

## 5 Conclusions

NDRG family genes played an important role in GC. The methylation of NDRG4 gene in PBLs was associated with GC risk, chemotherapy efficacy and prognosis. rs7202037 variations could affect the methylation level of NDRG4 gene. These findings provided a new direction for the screening of non-invasive markers of GC. These findings still needed to be validated by further research, so as to deepen the understanding of GC and improve the prognosis of GC patients.

## Data Availability

The datasets involved in this study can be obtained from the TCGA database, the public dataset GSE134520, and the TIMER database. The remaining data supporting the conclusions of this study will be made available upon reasonable request to the corresponding author. The authors will provide the original data supporting the conclusions of this article without reservation.

## Supplementary material

Supplementary Figure S1 NDRG4 and various immune cell types in gastric cancer. **(A)** The correlation coefficients between NDRG4 and various immune cells in gastric cancer. The vertical axis represents immune cells or indicators related to the tumor microenvironment, and the horizontal axis represents correlation coefficients. Different shapes and colors represent different cell types or indicator categories. **(B–I)** Scatter plots with linear regression lines showing the correlation between NDRG4 gene expression and the abundance of CAFs, endothelial cells, macrophages, stroma score, CD4^+^ T cells, and endothelial cells in gastric cancer. Each plot displays the correlation coefficient (*R*) and *P*-value.

Supplementary Figure S2 Prognostic and NDRG family genes in gastric cancer. **(A)** The survival difference between high and low expression of NDRG family genes in gastric cancer. Four panels represent disease-free interval (DFI), disease - specific survival (DSS), overall survival (OS), and progression -free interval (PFI), respectively. **(B, C)** Kaplan–Meier curve for DSS and PFI in gastric cancer patients stratified by NDRG4 expression (high/low). **(D, E)** Kaplan–Meier survival curves for DSS and PFI in gastric cancer patients, stratified by NDRG4 promoter methylation (high/low) and gene expression (high/low) groups.

Supplementary Figure S3 Nomogram for risk prediction of gastric cancer. **(A)** Nomogram for gastric cancer diagnosis. **(B)** Receiver operating characteristic curve (ROC). **(C)** Calibration curve. **(D)** Decision curve analysis (DCA).

Supplementary Figure S4 Nomogram for predicting the chemotherapy efficacy of in gastric cancer. **(A)** Nomogram for chemotherapy efficacy of gastric cancer. **(B)** Receiver operating characteristic curve (ROC). **(C)** Calibration curve. **(D)** Decision curve analysis (DCA).

Supplementary Figure S5 Nomogram for predicting the progress of in gastric cancer. **(A)** Nomogram for chemotherapy efficacy of gastric cancer. **(B)** Receiver operating characteristic curve (ROC). **(C)** Calibration curve. **(D)** Decision curve analysis (DCA).
