# Supplementary figures and images for: Association of NDRG4 gene methylation in peripheral blood leukocytes with gastric cancer risk, chemotherapy efficacy and prognosis
Source: Front Oncol. 2026 Apr 27;16:1778070. doi: 10.3389/fonc.2026.1778070 (PMC13158064; doi:10.3389/fonc.2026.1778070)

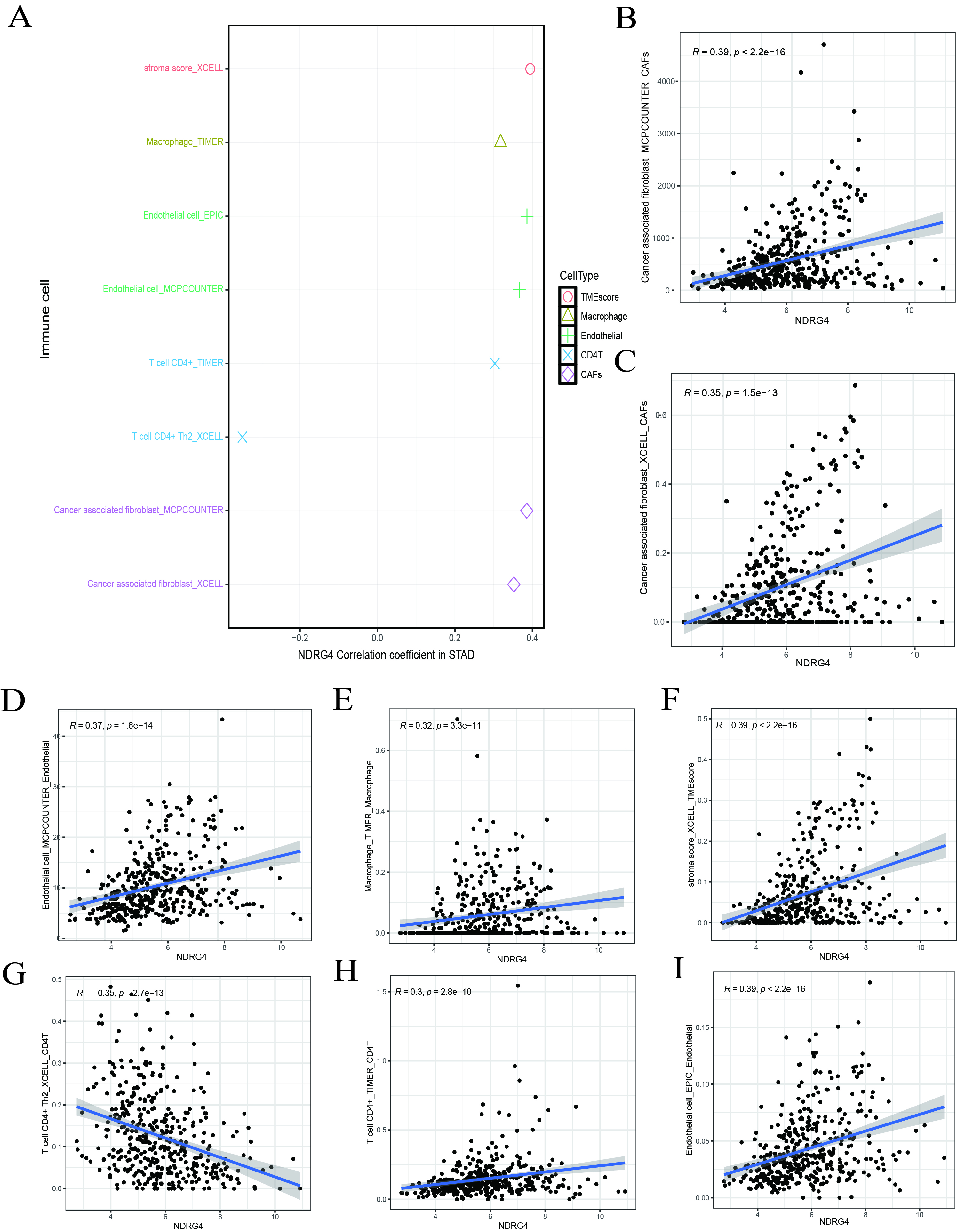

Supplement: Supplementary Figure S1 — NDRG4 and various immune cell types in gastric cancer. (A) The correlation coefficients between NDRG4 and various immune cells in gastric cancer. The vertical axis represents immune cells or indicators related to the tumor microenvironment, and the horizontal axis represents correlation coefficients. Different shapes and colors represent different cell types or indicator categories. (B–I) Scatter plots with linear regression lines showing the correlation between NDRG4 gene expression and the abundance of CAFs, endothelial cells, macrophages, stroma score, CD4+ T cells, and endothelial cells in gastric cancer. Each plot displays the correlation coefficient (R) and P-value. [file Image1.jpeg]

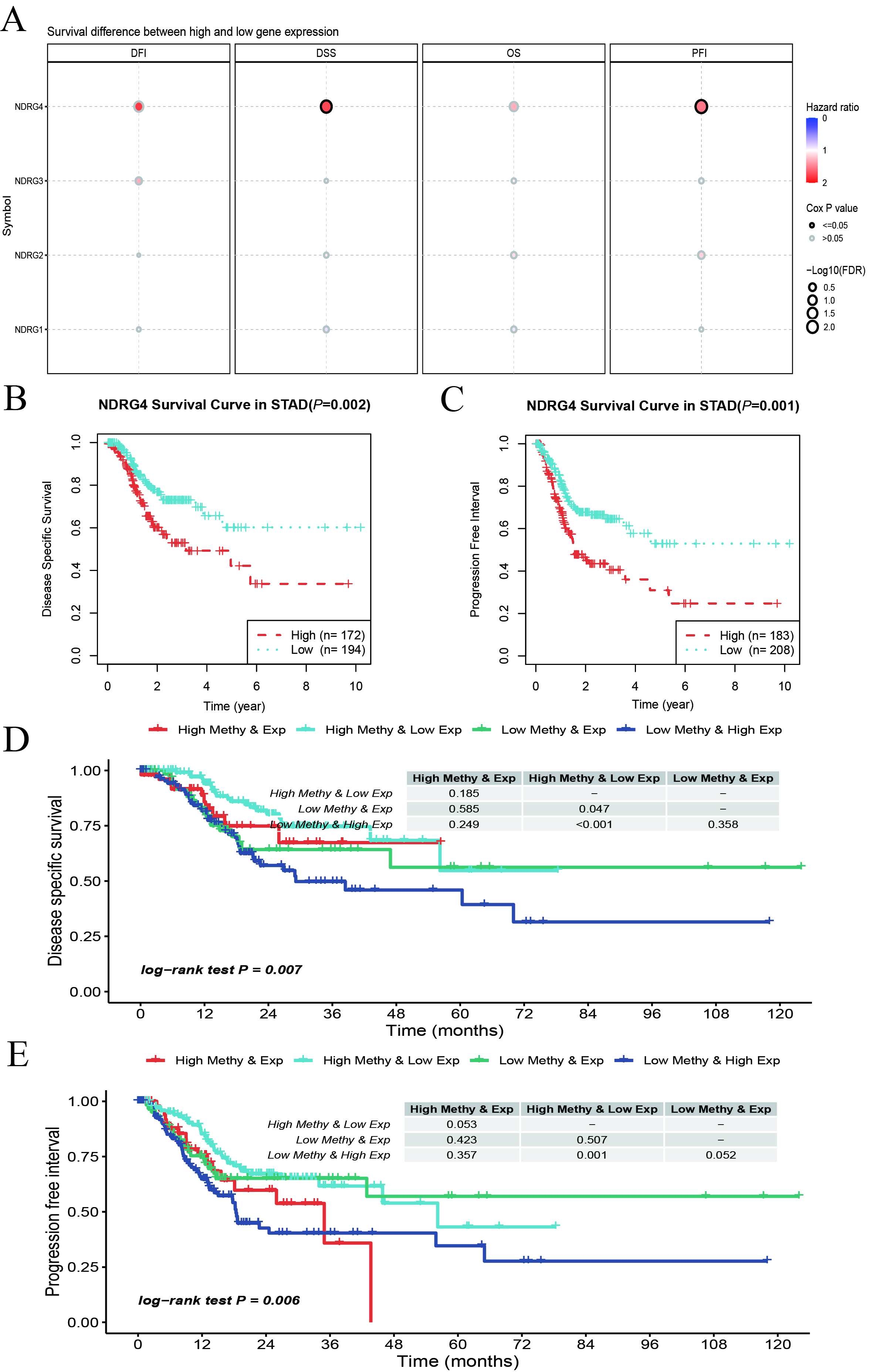

Supplement: Supplementary Figure S2 — Prognostic and NDRG family genes in gastric cancer. (A) The survival difference between high and low expression of NDRG family genes in gastric cancer. Four panels represent disease-free interval (DFI), disease - specific survival (DSS), overall survival (OS), and progression -free interval (PFI), respectively. (B, C) Kaplan–Meier curve for DSS and PFI in gastric cancer patients stratified by NDRG4 expression (high/low). (D, E) Kaplan–Meier survival curves for DSS and PFI in gastric cancer patients, stratified by NDRG4 promoter methylation (high/low) and gene expression (high/low) groups. [file Image2.jpeg]

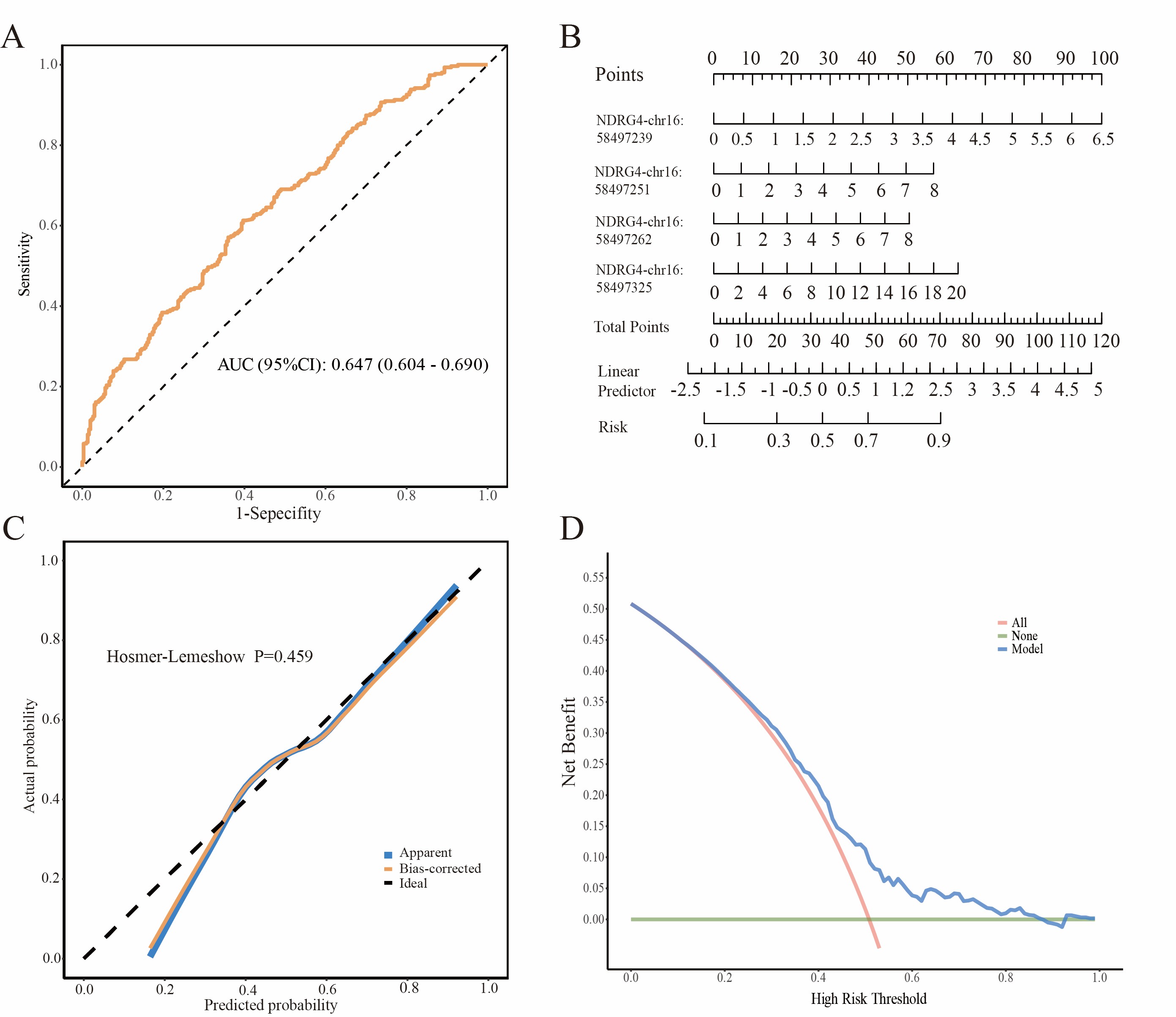

Supplement: Supplementary Figure S3 — Nomogram for risk prediction of gastric cancer. (A) Nomogram for gastric cancer diagnosis. (B) Receiver operating characteristic curve (ROC). (C) Calibration curve. (D) Decision curve analysis (DCA). [file Image3.jpeg]

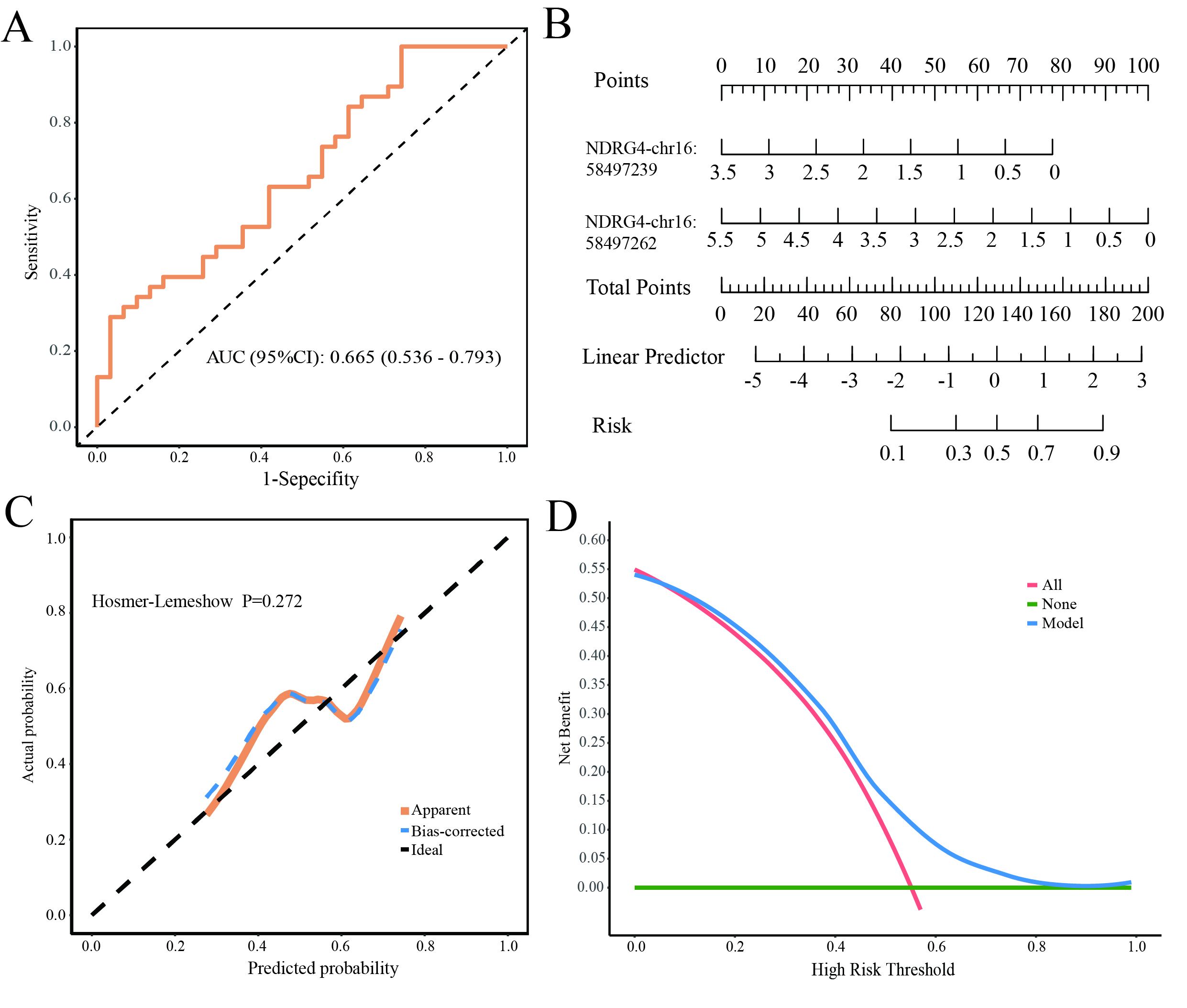

Supplement: Supplementary Figure S4 — Nomogram for predicting the chemotherapy efficacy of in gastric cancer. (A) Nomogram for chemotherapy efficacy of gastric cancer. (B) Receiver operating characteristic curve (ROC). (C) Calibration curve. (D) Decision curve analysis (DCA). [file Image4.jpeg]

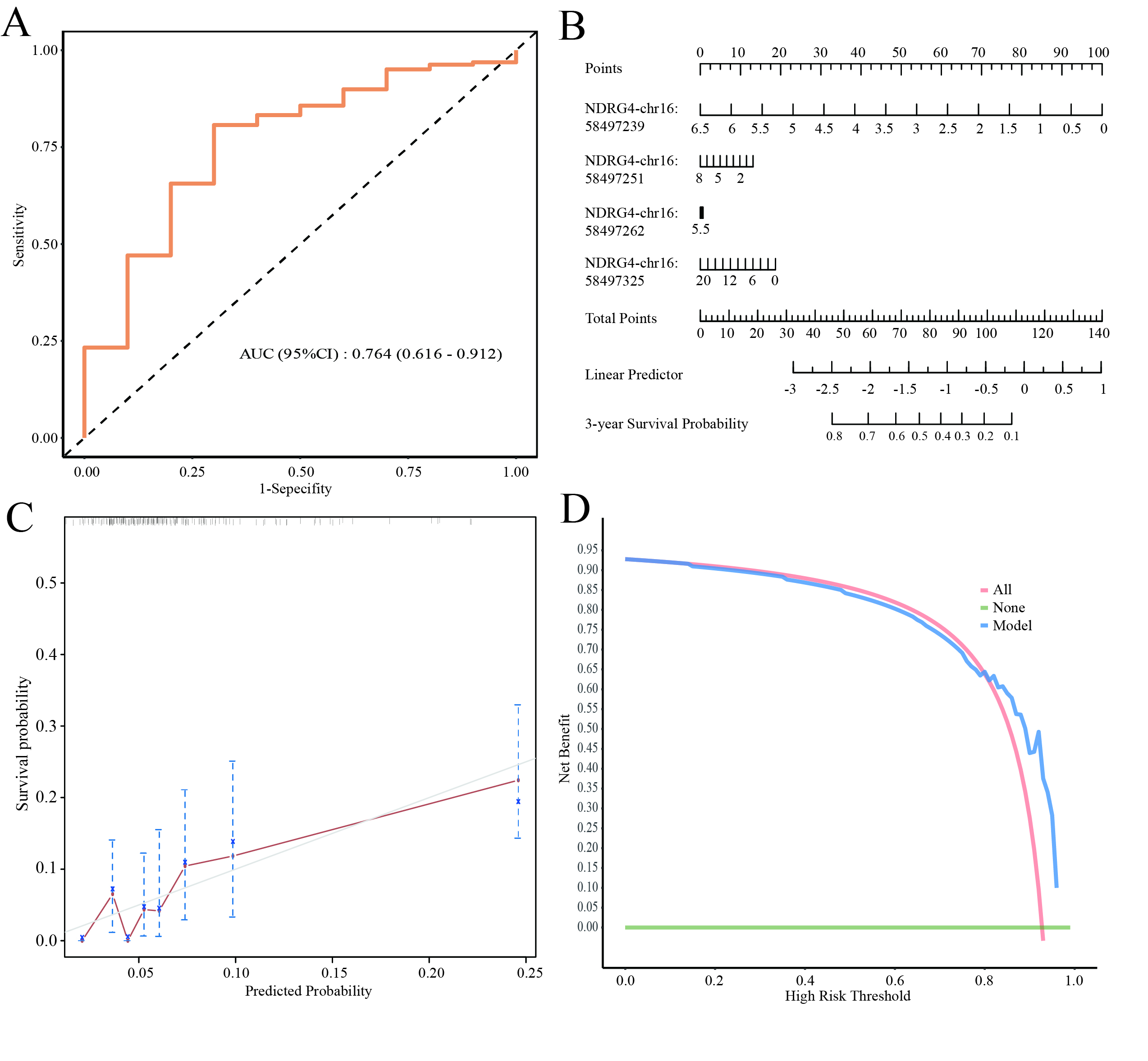

Supplement: Supplementary Figure S5 — Nomogram for predicting the progress of in gastric cancer. (A) Nomogram for chemotherapy efficacy of gastric cancer. (B) Receiver operating characteristic curve (ROC). (C) Calibration curve. (D) Decision curve analysis (DCA). [file Image5.jpeg]
