# Supplementary material for: Association of NDRG4 gene methylation in peripheral blood leukocytes with gastric cancer risk, chemotherapy efficacy and prognosis
Source: Front Oncol. 2026 Apr 27;16:1778070. doi: 10.3389/fonc.2026.1778070 (PMC13158064; doi:10.3389/fonc.2026.1778070)
Supplement: Supplementary file 6 [file Table1.docx]

Table S1 The positions of 17 CpG sites in the promoter region of NDRG4 gene

| **CpG sites** | **Position** | **Chromosome** | **Genome Position** |
| --- | --- | --- | --- |
| NDRG4-chr16:  58497230 | 61 | 16 | 58497230 |
| NDRG4-chr16:  58497236 | 67 | 16 | 58497236 |
| NDRG4-chr16:  58497239 | 70 | 16 | 58497239 |
| NDRG4-chr16:  58497251 | 82 | 16 | 58497251 |
| NDRG4-chr16:  58497259 | 90 | 16 | 58497259 |
| NDRG4-chr16:  58497262 | 93 | 16 | 58497262 |
| NDRG4-chr16:  58497265 | 96 | 16 | 58497265 |
| NDRG4-chr16:  58497267 | 98 | 16 | 58497267 |
| NDRG4-chr16:  58497269 | 100 | 16 | 58497269 |
| NDRG4-chr16:  58497292 | 123 | 16 | 58497292 |
| NDRG4-chr16:  58497304 | 135 | 16 | 58497304 |
| NDRG4-chr16:  58497309 | 140 | 16 | 58497309 |
| NDRG4-chr16:  58497325 | 156 | 16 | 58497325 |
| NDRG4-chr16:  58497327 | 158 | 16 | 58497327 |
| NDRG4-chr16:  58497329 | 160 | 16 | 58497329 |
| NDRG4-chr16:  58497332 | 163 | 16 | 58497332 |
| NDRG4-chr16:  58497337 | 168 | 16 | 58497337 |

Genome reference version: GRCh37. Genome Position: The position of this site on the reference genome.
