# Supplementary material for: Association of NDRG4 gene methylation in peripheral blood leukocytes with gastric cancer risk, chemotherapy efficacy and prognosis
Source: Front Oncol. 2026 Apr 27;16:1778070. doi: 10.3389/fonc.2026.1778070 (PMC13158064; doi:10.3389/fonc.2026.1778070)
Supplement: Supplementary file 7 [file Table2.docx]

**Table S2 Characteristic information of gastric cancer patients**

| Characteristic | **Preliminary Screening Stage** |  | **Validation Stage** |  | **Combination (Preliminary**+**Validation)** |
| --- | --- | --- | --- | --- | --- |
|  | **GC Patients**  **(n=100)** |  | **GC Patients**  **(n=210)** |  | **GC Patients**  **(n=310)** |
| **Degree of Differentiation** | | | | | |
| **Moderately and Well Differentiated** | 37 (37%) |  | 58 (27.62%) |  | 95 (30.65%) |
| **Poorly Differentiated** | 63 (63%) |  | 152 (72.38%) |  | 215 (69.35%) |
| TNM stage | | | | | |
| I-III | 41 (41%) |  | 84 (40%) |  | 125 (40.32%) |
| IV | 59 (59%) |  | 126 (60%) |  | 185 (59.67%) |
| **Tumor Location** | | | | | |
| **Gastric Antrum** | 27 (27%) |  | 62 (29.52%) |  | 89 (28.71%) |
| **Non-gastric Antrum** | 73 (73%) |  | 148 (70.48%) |  | 221 (71.29%) |
| **CEA Level** | | | | | |
| CEA <5 ng/ml | 58 (58%) |  | 134 (63.81%) |  | 192 (61.94%) |
| CEA ≥5 ng/ml | 42 (42%) |  | 76 (36.19%) |  | 118 (38.06%) |
| **CA199 Level** | | | | | |
| CA199 < 37 U/ml | 79 (79%) |  | 151 (71.90%) |  | 230 (74.19%) |
| CA199 ≥ 37 U/ml | 21 (21%) |  | 59 (28.10%) |  | 80 (25.81%) |
| **BMI Level (WHO)** | | | | | |
| < 18.5kg/m² | 5 (5%) |  | 10 (4.76%) |  | 15 (4.84%) |
| 18.5kg/m² ≤ BMI< 25.0kg/m² | 69 (69%) |  | 147 (70%) |  | 216 (69.68%) |
| 25.0kg/m² ≤ BMI<  30.0kg/m² | 20 (20%) |  | 47 (22.38%) |  | 67 (21.61%) |
| ≥30.0kg/m² | 6 (6%) |  | 6 (2.86%) |  | 12 (3.87%) |
| **Smoking status** | | | | | |
| No | 50 (50%) |  | 117 (55.71%) |  | 167 (53.87%) |
| **Yes** | 50 (50%) |  | 93 (44.29%) |  | 143 (46.13%) |
| **Alcohol consumption status** | | | | | |
| No | 53 (53%) |  | 104 (49.52%) |  | 157 (50.65%) |
| Yes | 47 (47%) |  | 106 (50.48%) |  | 153 (49.35%) |
