# Supplementary material for: Association of NDRG4 gene methylation in peripheral blood leukocytes with gastric cancer risk, chemotherapy efficacy and prognosis
Source: Front Oncol. 2026 Apr 27;16:1778070. doi: 10.3389/fonc.2026.1778070 (PMC13158064; doi:10.3389/fonc.2026.1778070)
Supplement: Supplementary file 8 [file Table3.docx]

Table S3 Association between methylation of NDRG4 gene/sites and gastric cancer differentiation degree

| Gene/Sites | Methylation level^a^ | |  | Logistic regression analysis | | | | |
| --- | --- | --- | --- | --- | --- | --- | --- | --- |
|  | **Moderately and Well Differentiated** | **Poorly Differentiated** |  | Crude *OR*(95%*CI*) | Crude *P-*value | Adjusted *OR* (95%*CI*)^*^ | Adjusted *P*-value^*^ | *P*_BH_ |
| NDRG4-gene | 1.72(1.45,2.04) | 1.58(1.39,1.85) |  | 0.480(0.265-0.869) | 0.015 | 0.509(0.275-0.941) | 0.031 | 0.226 |
| NDRG4-chr16:  58497230 | 2.28(1.73,3.13) | 2.04(1.63,2.75) |  | 0.792(0.628-1.000) | 0.050 | 0.795(0.628-1.007) | 0.057 | 0.226 |
| NDRG4-chr16:  58497236 | 1.61(1.20,2.04) | 1.52(1.16,1.94) |  | 0.970(0.690-1.364) | 0.862 | 0.969(0.686-1.367) | 0.856 | 0.856 |
| NDRG4-chr16:  58497239 | 0.95(0.73,1.30) | 0.91(0.73,1.11) |  | 0.870(0.582-1.301) | 0.497 | 0.897(0.598-1.344) | 0.597 | 0.774 |
| NDRG4-chr16:  58497251 | 0.65(0.39,0.90) | 0.57(0.45,0.78) |  | 0.592(0.343-1.023) | 0.060 | 0.621(0.361-1.068) | 0.085 | 0.226 |
| NDRG4-chr16:  58497259 | 0.99(0.71,1.30) | 0.97(0.77,1.20) |  | 0.767(0.505-1.166) | 0.214 | 0.819(0.535-1.255) | 0.360 | 0.643 |
| NDRG4-chr16:  58497262 | 1.15(0.94,1.47) | 0.99(0.76,1.27) |  | 0.643(0.404-1.025) | 0.063 | 0.662(0.412-1.063) | 0.088 | 0.226 |
| NDRG4-chr16:  58497265 | 1.40(1.13,1.69) | 1.25(0.99,1.56) |  | 0.603(0.377-0.964) | 0.035 | 0.605(0.377-0.971) | 0.037 | 0.226 |
| NDRG4-chr16:  58497267 | 0.95(0.74,1.12) | 0.93(0.73,1.14) |  | 0.958(0.512-1.792) | 0.892 | 0.943(0.500-1.779) | 0.856 | 0.856 |
| NDRG4-chr16:  58497269 | 1.00(0.73,1.34) | 1.02(0.76,1.36) |  | 1.030(0.708-1.499) | 0.876 | 1.095(0.745-1.608) | 0.645 | 0.774 |
| NDRG4-chr16:  58497292 | 1.55(1.19,2.02) | 1.49(1.12,1.90) |  | 0.839(0.597-1.181) | 0.314 | 0.861(0.610-1.214) | 0.393 | 0.643 |
| NDRG4-chr16:  58497304 | 1.80(1.40,2.19) | 1.60(1.30,2.02) |  | 0.759(0.521-1.105) | 0.150 | 0.780(0.531-1.145) | 0.204 | 0.408 |
| NDRG4-chr16:  58497309 | 1.97(1.55,2.78) | 1.88(1.45,2.29) |  | 0.774(0.583-1.027) | 0.076 | 0.788(0.593-1.049) | 0.102 | 0.230 |
| NDRG4-chr16:  58497325 | 3.19(2.62,3.95) | 3.01(2.50,3.57) |  | 0.826(0.682-0.999) | 0.049 | 0.848(0.701-1.025) | 0.088 | 0.226 |
| NDRG4-chr16:  58497327 | 1.49(1.16,1.89) | 1.46(1.17,1.88) |  | 0.891(0.633-1.254) | 0.508 | 0.941(0.659-1.345) | 0.740 | 0.833 |
| NDRG4-chr16:  58497329 | 1.89(1.40,2.41) | 1.83(1.39,2.31) |  | 0.905(0.664-1.234) | 0.529 | 0.921(0.672-1.262) | 0.610 | 0.774 |
| NDRG4-chr16:  58497332 | 3.74(3.20,4.65) | 3.56(3.08,4.40) |  | 0.813(0.667-0.991) | 0.041 | 0.835(0.682-1.021) | 0.079 | 0.226 |
| NDRG4-chr16:  58497337 | 1.67(1.26,2.22) | 1.62(1.24,2.03) |  | 0.903(0.669-1.218) | 0.504 | 0.908(0.671-1.228) | 0.531 | 0.774 |

^a^ Methylation level is expressed as a percentage, data was expressed as median (*P*_25_, *P*_75_). ^*^Adjusted for age and sex. *OR*: odds ratio. BH: **Benjamini-Hochberg.**
