# Supplementary material for: Association of NDRG4 gene methylation in peripheral blood leukocytes with gastric cancer risk, chemotherapy efficacy and prognosis
Source: Front Oncol. 2026 Apr 27;16:1778070. doi: 10.3389/fonc.2026.1778070 (PMC13158064; doi:10.3389/fonc.2026.1778070)
Supplement: Supplementary file 9 [file Table4.docx]

Table S4 Association between methylation of the NDRG4 gene/sites and the TNM stage

| Gene/Sites | Methylation level^a^ | |  | Logistic regression analysis | | | | |
| --- | --- | --- | --- | --- | --- | --- | --- | --- |
|  | Stage I-III | **Stage IV** |  | Crude *OR*(95%*CI*) | Crude *P-*value | Adjusted *OR* (95%*CI*)^*^ | Adjusted *P*-value^*^ | *P*_BH_ |
| NDRG4-gene | 1.61(1.44,1.96) | 1.61(1.38,1.86) |  | 0.822(0.468-1.445) | 0.496 | 0.701(0.388-1.264) | 0.237 | 0.723 |
| NDRG4-chr16:  58497230 | 2.15(1.71,3.14) | 2.07(1.62,2.78) |  | 0.901(0.722-1.124) | 0.354 | 0.871(0.695-1.093) | 0.234 | 0.723 |
| NDRG4-chr16:  58497236 | 1.54(1.14,1.95) | 1.57(1.19,1.99) |  | 0.984(0.714-1.358) | 0.924 | 0.973(0.704-1.344) | 0.868 | 0.938 |
| NDRG4-chr16:  58497239 | 0.96(0.77,1.20) | 0.90(0.71,1.14) |  | 0.959(0.649-1.417) | 0.834 | 0.954(0.643-1.417) | 0.817 | 0.938 |
| NDRG4-chr16:  58497251 | 0.66(0.46,0.88) | 0.54(0.42,0.75) |  | 0.851(0.564-1.285) | 0.443 | 0.856(0.566-1.294) | 0.460 | 0.920 |
| NDRG4-chr16:  58497259 | 0.93(0.68,1.24) | 1.00(0.79,1.25) |  | 1.340(0.856-2.100) | 0.201 | 1.272(0.809-2.002) | 0.297 | 0.729 |
| NDRG4-chr16:  58497262 | 1.07(0.86,1.37) | 1.03(0.76,1.35) |  | 0.922(0.596-1.426) | 0.714 | 0.891(0.573-1.386) | 0.609 | 0.938 |
| NDRG4-chr16:  58497265 | 1.34(1.06,1.61) | 1.24(1.01,1.59) |  | 0.868(0.562-1.342) | 0.525 | 0.800(0.513-1.247) | 0.324 | 0.729 |
| NDRG4-chr16:  58497267 | 0.93(0.71,1.16) | 0.93(0.74,1.12) |  | 1.042(0.577-1.881) | 0.891 | 1.026(0.567-1.856) | 0.932 | 0.938 |
| NDRG4-chr16:  58497269 | 1.01(0.74,1.39) | 1.00(0.74,1.30) |  | 1.078(0.755-1.540) | 0.678 | 1.034(0.723-1.479) | 0.856 | 0.938 |
| NDRG4-chr16:  58497292 | 1.44(1.11,1.90) | 1.54(1.17,1.93) |  | 1.050(0.755-1.460) | 0.774 | 1.013(0.727-1.413) | 0.938 | 0.938 |
| NDRG4-chr16:  58497304 | 1.71(1.35,2.09) | 1.67(1.30,2.06) |  | 0.879(0.617-1.252) | 0.476 | 0.803(0.557-1.158) | 0.241 | 0.723 |
| NDRG4-chr16:  58497309 | 1.94(1.50,2.38) | 1.84(1.49,2.36) |  | 0.995(0.759-1.304) | 0.970 | 0.958(0.728-1.260) | 0.758 | 0.938 |
| NDRG4-chr16:  58497325 | 3.13(2.62,3.97) | 2.98(2.50,3.54) |  | 0.928(0.789-1.092) | 0.370 | 0.898(0.753-1.071) | 0.230 | 0.723 |
| NDRG4-chr16:  58497327 | 1.49(1.14,1.89) | 1.45(1.20,1.89) |  | 0.858(0.618-1.191) | 0.360 | 0.766(0.541-1.085) | 0.134 | 0.723 |
| NDRG4-chr16:  58497329 | 1.85(1.40,2.43) | 1.86(1.39,2.31) |  | 0.988(0.736-1.325) | 0.933 | 0.932(0.690-1.259) | 0.647 | 0.938 |
| NDRG4-chr16:  58497332 | 3.66(3.19,4.62) | 3.58(3.05,4.38) |  | 0.911(0.755-1.099) | 0.331 | 0.886(0.731-1.074) | 0.217 | 0.723 |
| NDRG4-chr16:  58497337 | 1.63(1.27,2.17) | 1.57(1.22,2.05) |  | 0.980(0.737-1.304) | 0.892 | 0.943(0.705-1.261) | 0.692 | 0.938 |

^a^ Methylation level is expressed as a percentage, data was expressed as median (*P*_25_, *P*_75_). ^*^Adjusted for age and sex. *OR*: odds ratio. BH: **Benjamini-Hochberg.**
