# Supplementary material for: Association of NDRG4 gene methylation in peripheral blood leukocytes with gastric cancer risk, chemotherapy efficacy and prognosis
Source: Front Oncol. 2026 Apr 27;16:1778070. doi: 10.3389/fonc.2026.1778070 (PMC13158064; doi:10.3389/fonc.2026.1778070)
Supplement: Supplementary file 10 [file Table5.docx]

Table S5 Association between methylation of the NDRG4 gene/sites and tumor location

| Gene/Sites | Methylation level^a^ | |  | Logistic regression analysis | | | | |
| --- | --- | --- | --- | --- | --- | --- | --- | --- |
|  | **Non-gastric Antrum** | **Gastric Antrum** |  | Crude *OR*(95%*CI*) | Crude *P-*value | Adjusted *OR* (95%*CI*)^*^ | Adjusted *P*-value^*^ | *P*_BH_ |
| NDRG4-gene | 1.60(1.41,1.89) | 1.63(1.39,1.95) |  | 1.117(0.608-2.053) | 0.721 | 1.276(0.678-2.403) | 0.450 | 0.822 |
| NDRG4-chr16:  58497230 | 2.05(1.64,2.72) | 2.18(1.68,3.24) |  | 1.214(0.959-1.535) | 0.107 | 1.246(0.981-1.584) | 0.072 | 0.774 |
| NDRG4-chr16:  58497236 | 1.54(1.15,1.98) | 1.57(1.21,1.98) |  | 1.054(0.746-1.489) | 0.767 | 1.061(0.751-1.497) | 0.738 | 0.822 |
| NDRG4-chr16:  58497239 | 0.91(0.71,1.15) | 0.93(0.80,1.26) |  | 1.090(0.722-1.646) | 0.682 | 1.108(0.733-1.675) | 0.627 | 0.822 |
| NDRG4-chr16:  58497251 | 0.58(0.45,0.82) | 0.59(0.43,0.86) |  | 0.916(0.567-1.479) | 0.718 | 0.932(0.576-1.509) | 0.776 | 0.822 |
| NDRG4-chr16:  58497259 | 0.99(0.77,1.27) | 0.99(0.73,1.19) |  | 0.780(0.476-1.277) | 0.323 | 0.822(0.498-1.358) | 0.445 | 0.822 |
| NDRG4-chr16:  58497262 | 1.05(0.82,1.30) | 1.08(0.73,1.47) |  | 1.347(0.849-2.137) | 0.207 | 1.405(0.881-2.239) | 0.153 | 0.822 |
| NDRG4-chr16:  58497265 | 1.30(1.03,1.60) | 1.26(1.01,1.59) |  | 1.000(0.624-1.602) | 0.999 | 1.045(0.647-1.688) | 0.856 | 0.856 |
| NDRG4-chr16:  58497267 | 0.95(0.75,1.15) | 0.89(0.72,1.09) |  | 0.760(0.397-1.457) | 0.409 | 0.763(0.400-1.458) | 0.413 | 0.822 |
| NDRG4-chr16:  58497269 | 1.01(0.74,1.32) | 1.00(0.78,1.38) |  | 1.008(0.694-1.466) | 0.965 | 1.058(0.725-1.543) | 0.769 | 0.822 |
| NDRG4-chr16:  58497292 | 1.53(1.17,1.93) | 1.48(1.12,1.90) |  | 0.824(0.565-1.201) | 0.314 | 0.848(0.580-1.239) | 0.393 | 0.822 |
| NDRG4-chr16:  58497304 | 1.69(1.31,2.06) | 1.65(1.36,2.11) |  | 1.081(0.737-1.584) | 0.690 | 1.153(0.777-1.712) | 0.479 | 0.822 |
| NDRG4-chr16:  58497309 | 1.89(1.49,2.32) | 1.92(1.48,2.53) |  | 1.057(0.791-1.413) | 0.706 | 1.091(0.813-1.464) | 0.563 | 0.822 |
| NDRG4-chr16:  58497325 | 3.06(2.56,3.66) | 2.93(2.44,3.58) |  | 0.917(0.750-1.122) | 0.399 | 0.943(0.774-1.150) | 0.563 | 0.822 |
| NDRG4-chr16:  58497327 | 1.46(1.15,1.86) | 1.50(1.22,1.95) |  | 1.044(0.734-1.485) | 0.811 | 1.134(0.786-1.635) | 0.502 | 0.822 |
| NDRG4-chr16:  58497329 | 1.79(1.34,2.35) | 1.91(1.50,2.31) |  | 1.264(0.923-1.732) | 0.145 | 1.327(0.961-1.833) | 0.086 | 0.774 |
| NDRG4-chr16:  58497332 | 3.57(3.14,4.46) | 3.79(3.09,4.59) |  | 1.095(0.896-1.337) | 0.375 | 1.131(0.921-1.388) | 0.242 | 0.822 |
| NDRG4-chr16:  58497337 | 1.63(1.26,2.06) | 1.63(1.22,2.16) |  | 0.931(0.679-1.277) | 0.658 | 0.952(0.692-1.309) | 0.761 | 0.822 |

^a^ Methylation level is expressed as a percentage, data was expressed as median (*P*_25_, *P*_75_). ^*^Adjusted for age and sex. *OR*: odds ratio. BH: **Benjamini-Hochberg.**
