# Supplementary material for: Association of NDRG4 gene methylation in peripheral blood leukocytes with gastric cancer risk, chemotherapy efficacy and prognosis
Source: Front Oncol. 2026 Apr 27;16:1778070. doi: 10.3389/fonc.2026.1778070 (PMC13158064; doi:10.3389/fonc.2026.1778070)
Supplement: Supplementary file 11 [file Table6.docx]

Table S6 Association between methylation of the NDRG4 gene/sites and CEA level

| Gene/Sites | Methylation level^a^ | |  | Logistic regression analysis | | | | |
| --- | --- | --- | --- | --- | --- | --- | --- | --- |
|  | CEA< 5ng/ml | CEA≥5ng/ml |  | Crude *OR*(95%*CI*) | Crude *P-*value | Adjusted *OR* (95%*CI*)^*^ | Adjusted *P*-value^*^ | *P*_BH_ |
| NDRG4-gene | 1.63(1.42,1.96) | 1.60(1.39,1.84) |  | 0.615(0.337-1.122) | 0.113 | 0.515(0.275-0.964) | 0.038 | 0.252 |
| NDRG4-chr16:  58497230 | 2.12(1.68,2.92) | 2.08(1.56,2.68) |  | 0.825(0.652-1.044) | 0.109 | 0.802(0.632-1.018) | 0.070 | 0.252 |
| NDRG4-chr16:  58497236 | 1.58(1.17,1.98) | 1.54(1.18,1.98) |  | 0.958(0.691-1.330) | 0.799 | 0.949(0.681-1.324) | 0.759 | 0.804 |
| NDRG4-chr16:  58497239 | 0.95(0.73,1.24) | 0.90(0.72,1.08) |  | 0.874(0.574-1.330) | 0.529 | 0.851(0.550-1.315) | 0.467 | 0.543 |
| NDRG4-chr16:  58497251 | 0.64(0.46,0.89) | 0.52(0.41,0.72) |  | 0.778(0.465-1.300) | 0.337 | 0.761(0.452-1.283) | 0.306 | 0.531 |
| NDRG4-chr16:  58497259 | 0.99(0.74,1.28) | 0.97(0.80,1.19) |  | 0.916(0.601-1.396) | 0.684 | 0.855(0.556-1.313) | 0.473 | 0.543 |
| NDRG4-chr16:  58497262 | 1.08(0.82,1.37) | 1.00(0.76,1.35) |  | 0.759(0.474-1.214) | 0.249 | 0.733(0.459-1.171) | 0.194 | 0.424 |
| NDRG4-chr16:  58497265 | 1.34(1.03,1.65) | 1.23(1.01,1.52) |  | 0.697(0.445-1.093) | 0.116 | 0.650(0.410-1.032) | 0.068 | 0.252 |
| NDRG4-chr16:  58497267 | 0.95(0.76,1.16) | 0.89(0.71,1.07) |  | 0.533(0.285-0.996) | 0.048 | 0.519(0.275-0.981) | 0.043 | 0.252 |
| NDRG4-chr16:  58497269 | 1.03(0.74,1.43) | 0.99(0.74,1.24) |  | 0.885(0.611-1.283) | 0.520 | 0.833(0.566-1.226) | 0.354 | 0.531 |
| NDRG4-chr16:  58497292 | 1.52(1.17,1.92) | 1.50(1.11,1.96) |  | 1.014(0.729-1.411) | 0.934 | 0.980(0.701-1.370) | 0.906 | 0.906 |
| NDRG4-chr16:  58497304 | 1.69(1.37,2.11) | 1.66(1.26,2.03) |  | 0.733(0.508-1.057) | 0.096 | 0.673(0.461-0.981) | 0.040 | 0.252 |
| NDRG4-chr16:  58497309 | 1.96(1.49,2.48) | 1.78(1.46,2.18) |  | 0.851(0.641-1.131) | 0.267 | 0.818(0.611-1.093) | 0.174 | 0.424 |
| NDRG4-chr16:  58497325 | 3.13(2.53,3.66) | 2.91(2.51,3.50) |  | 0.961(0.812-1.139) | 0.647 | 0.929(0.774-1.115) | 0.429 | 0.543 |
| NDRG4-chr16:  58497327 | 1.50(1.15,1.87) | 1.48(1.20,1.93) |  | 0.924(0.659-1.295) | 0.645 | 0.839(0.587-1.199) | 0.336 | 0.531 |
| NDRG4-chr16:  58497329 | 1.83(1.41,2.38) | 1.91(1.34,2.30) |  | 0.939(0.697-1.267) | 0.681 | 0.896(0.660-1.217) | 0.483 | 0.543 |
| NDRG4-chr16:  58497332 | 3.66(3.09,4.56) | 3.60(3.14,4.20) |  | 0.907(0.747-1.102) | 0.328 | 0.879(0.721-1.071) | 0.201 | 0.424 |
| NDRG4-chr16:  58497337 | 1.64(1.25,2.21) | 1.58(1.25,1.98) |  | 0.853(0.633-1.149) | 0.296 | 0.825(0.609-1.117) | 0.212 | 0.424 |

^a^ Methylation level is expressed as a percentage, data was expressed as median (*P*_25_, *P*_75_). ^*^Adjusted for age and sex. *OR*: odds ratio. BH: **Benjamini-Hochberg.**
