# Supplementary material for: Association of NDRG4 gene methylation in peripheral blood leukocytes with gastric cancer risk, chemotherapy efficacy and prognosis
Source: Front Oncol. 2026 Apr 27;16:1778070. doi: 10.3389/fonc.2026.1778070 (PMC13158064; doi:10.3389/fonc.2026.1778070)
Supplement: Supplementary file 12 [file Table7.docx]

Table S7 Association between methylation of the NDRG4 gene/sites and CA199 level

| Gene/Sites | Methylation level^a^ | |  | Logistic regression analysis | | | | |
| --- | --- | --- | --- | --- | --- | --- | --- | --- |
|  | CA199<37U/ml | CA199≥37U/ml |  | Crude *OR*(95%*CI*) | Crude *P-*value | Adjusted *OR* (95%*CI*)^*^ | Adjusted *P*-value^*^ | *P*_BH_ |
| NDRG4-gene | 1.64(1.44,1.93) | 1.53(1.37,1.81) |  | 0.612(0.309-1.209) | 0.157 | 0.543(0.268-1.103) | 0.091 | 0.331 |
| NDRG4-chr16:  58497230 | 2.17(1.68,2.92) | 1.96(1.58,2.61) |  | 0.868(0.669-1.127) | 0.289 | 0.853(0.656-1.109) | 0.235 | 0.406 |
| NDRG4-chr16:  58497236 | 1.54(1.14,1.96) | 1.58(1.22,2.09) |  | 1.204(0.848-1.710) | 0.299 | 1.200(0.843-1.706) | 0.311 | 0.406 |
| NDRG4-chr16:  58497239 | 0.94(0.75,1.20) | 0.87(0.70,1.08) |  | 0.748(0.439-1.273) | 0.284 | 0.732(0.425-1.263) | 0.263 | 0.406 |
| NDRG4-chr16:  58497251 | 0.60(0.46,0.85) | 0.56(0.42,0.75) |  | 1.049(0.679-1.620) | 0.831 | 1.050(0.678-1.626) | 0.829 | 0.829 |
| NDRG4-chr16:  58497259 | 0.95(0.71,1.20) | 1.05(0.83,1.26) |  | 1.426(0.927-2.195) | 0.106 | 1.402(0.902-2.179) | 0.133 | 0.399 |
| NDRG4-chr16:  58497262 | 1.07(0.83,1.37) | 0.89(0.73,1.23) |  | 0.540(0.302-0.966) | 0.038 | 0.532(0.297-0.951) | 0.033 | 0.297 |
| NDRG4-chr16:  58497265 | 1.30(1.04,1.60) | 1.23(0.99,1.57) |  | 0.811(0.495-1.329) | 0.406 | 0.773(0.467-1.279) | 0.316 | 0.406 |
| NDRG4-chr16:  58497267 | 0.95(0.73,1.15) | 0.92(0.73,1.05) |  | 0.842(0.432-1.642) | 0.613 | 0.831(0.423-1.633) | 0.591 | 0.665 |
| NDRG4-chr16:  58497269 | 1.05(0.74,1.41) | 0.94(0.74,1.23) |  | 0.815(0.522-1.274) | 0.370 | 0.783(0.494-1.241) | 0.298 | 0.406 |
| NDRG4-chr16:  58497292 | 1.53(1.19,1.92) | 1.47(0.99,1.93) |  | 0.767(0.514-1.145) | 0.195 | 0.745(0.495-1.120) | 0.157 | 0404 |
| NDRG4-chr16:  58497304 | 1.71(1.37,2.11) | 1.58(1.28,2.00) |  | 0.741(0.493-1.114) | 0.149 | 0.698(0.460-1.060) | 0.092 | 0.331 |
| NDRG4-chr16:  58497309 | 1.92(1.51,2.42) | 1.78(1.37,2.11) |  | 0.665(0.468-0.946) | 0.023 | 0.640(0.447-0.917) | 0.015 | 0.270 |
| NDRG4-chr16:  58497325 | 3.07(2.51,3.73) | 2.88(2.53,3.50) |  | 0.991(0.826-1.189) | 0.920 | 0.974(0.804-1.181) | 0.791 | 0.829 |
| NDRG4-chr16:  58497327 | 1.50(1.16,1.88) | 1.43(1.20,1.95) |  | 0.942(0.646-1.372) | 0.754 | 0.886(0.596-1.319) | 0.552 | 0.662 |
| NDRG4-chr16:  58497329 | 1.89(1.42,2.36) | 1.68(1.33,2.28) |  | 0.757(0.534-1.073) | 0.118 | 0.726(0.508-1.037) | 0.078 | 0.331 |
| NDRG4-chr16:  58497332 | 3.66(3.15,4.51) | 3.56(3.00,4.18) |  | 0.895(0.718-1.115) | 0.322 | 0.879(0.703-1.099) | 0.257 | 0.406 |
| NDRG4-chr16:  58497337 | 1.64(1.26,2.17) | 1.50(1.16,1.97) |  | 0.818(0.582-1.149) | 0.247 | 0.797(0.565-1.124) | 0.196 | 0.406 |

^a^ Methylation level is expressed as a percentage, data was expressed as median (*P*_25_, *P*_75_). ^*^Adjusted for age and sex. *OR*: odds ratio. BH: **Benjamini-Hochberg.**
