# Supplementary material for: Association of NDRG4 gene methylation in peripheral blood leukocytes with gastric cancer risk, chemotherapy efficacy and prognosis
Source: Front Oncol. 2026 Apr 27;16:1778070. doi: 10.3389/fonc.2026.1778070 (PMC13158064; doi:10.3389/fonc.2026.1778070)
Supplement: Supplementary file 13 [file Table8.docx]

### Table S8 Association between NDRG4 CpG sites methylation and chemotherapy efficacy of gastric cancer

| Sites | Methylation level^a^ | |  | Logistic regression analysis | | | | | *P*_BH_ |
| --- | --- | --- | --- | --- | --- | --- | --- | --- | --- |
|  | PD | nPD |  | Crude *OR(*95%*CI)* | Crude *P-*value | | Adjusted *OR* (95%*CI*)^*^ | Adjusted *P*-value^*^ |  |
| NDRG4-chr16:  58497239 | 0.88(0.72,1.14) | 1.09(0.76,1.58) |  | 0.280(0.095-0.823) | 0.021 | 0.238(0.070-0.805) | | **0.021** | **0.033** |
| NDRG4-chr16:  58497262 | 0.95(0.57,1.29) | 1.14(0.89,1.47) |  | 0.334(0.120-0.933) | 0.036 | 0.299(0.099-0.908) | | **0.033** | **0.033** |

^a^ Methylation level is expressed as a percentage, data was expressed as median (*P*_25_, *P*_75_). ^*^Adjusted for age, sex, smoking, drinking and differentiation degree. PD: **progressive disease.** nPD: including complete response, partial response, and stable disease. *CI*: confidence interval. *OR*: odds ratio. BH: **Benjamini-Hochberg.**
