# Supplementary material for: Association of NDRG4 gene methylation in peripheral blood leukocytes with gastric cancer risk, chemotherapy efficacy and prognosis
Source: Front Oncol. 2026 Apr 27;16:1778070. doi: 10.3389/fonc.2026.1778070 (PMC13158064; doi:10.3389/fonc.2026.1778070)
Supplement: Supplementary file 14 [file Table9.docx]

### Table S9 Association between NDRG4 methylation sites and prognosis of gastric cancer patients

| Sites | Methylation level^a^ | |  | Univariate cox regression model | | Multivariate cox regression model^*^ | | *P*_BH_ |
| --- | --- | --- | --- | --- | --- | --- | --- | --- |
|  | nPD/Non-recurrence/Alive | PD/Recurrence /Dead |  | *HR(95% CI)* | *P* value | *HR^*^*(95% *CI*) | *P^*^-*value |  |
| DFS | | | | | | | | |
| NDRG4-chr16:58497239 | 0.97(0.77,1.32) | 0.95(0.77,1.17) |  | 0.923 (0.597 - 1.427) | 0.719 | 0.875 (0.545 - 1.404) | 0.580 | 0.680 |
| NDRG4-chr16:58497262 | 1.14(0.91,1.36) | 1.03(0.85,1.37) |  | 1.074 (0.641 - 1.798) | 0.786 | 1.123 (0.646 - 1.952) | 0.680 | 0.680 |
| PFS | | | | | | | | |
| NDRG4-chr16:58497239 | 1.50(1.24,1.82) | 0.88(0.70,1.06) |  | 0.558 (0.382 - 0.814) | 0.003 | 0.581 (0.391 - 0.856) | 0.008 | **0.016** |
| NDRG4-chr16:58497262 | 1.36(1.01,1.43) | 1.01(0.76,1.25) |  | 0.853 (0.623 - 1.167) | 0.320 | 0.850 (0.609 - 1.171) | 0.331 | 0.331 |
| OS | | | | | | | | |
| NDRG4-chr16:58497239 | 1.10(0.83,1.54) | 0.88(0.71,1.10) |  | 0.908 (0.719 - 1.148) | 0.422 | 0.923 (0.740 - 1.150) | 0.474 | 0.946 |
| NDRG4-chr16:58497262 | 1.16(0.89,1.41) | 1.02(0.79,1.30) |  | 1.017 (0.778 - 1.330) | 0.901 | 1.009 (0.775 - 1.315) | 0.946 | 0.946 |

^a^ Methylation level is expressed as a percentage, data was expressed as median (*P*_25_, *P*_75_). ^*^DFS: Adjusted for age, gender, smoking, drinking, differentiation degree and TNM stage. PFS: Adjusted for age, gender, smoking, drinking, differentiation degree. OS: Adjusted for age, gender, smoking, drinking, differentiation degree and TNM stage. PFS: progression-free survival. DFS: disease-free survival. OS: overall survival. PD: **progressive disease.** nPD: including complete response, partial response, and stable disease. *CI*: confidence interval. *HR*: hazard ratio. BH: **Benjamini-Hochberg.**
