# Supplementary material for: Association of NDRG4 gene methylation in peripheral blood leukocytes with gastric cancer risk, chemotherapy efficacy and prognosis
Source: Front Oncol. 2026 Apr 27;16:1778070. doi: 10.3389/fonc.2026.1778070 (PMC13158064; doi:10.3389/fonc.2026.1778070)
Supplement: Supplementary file 15 [file Table10.docx]

Table S10 Association between methylation of NDRG4 gene and SNP genotypes under different genetic models

| Model | NDRG4-chr16:  58497239 | | |  | NDRG4-chr16:  58497325 | | |
| --- | --- | --- | --- | --- | --- | --- | --- |
|  | Statistic | *P-*value | *P*_BH_ |  | Statistic | *P-*value | *P*_BH_ |
| Dominant model  （AA vs. AC+CC） | -0.570 | 0.568 | 0.568 |  | -2.192 | 0.028 | **0.028** |
| Recessive model  （AA+AC vs. CC） | -2.655 | 0.008 | **0.024** |  | -2.725 | 0.006 | **0.011** |
| Additive model  （AA/AC/CC） | 7.057 | 0.029 | **0.044** |  | 9.907 | 0.007 | **0.011** |

Comparison between groups used Mann-Whitney/Kruskal-Wallis test. BH: **Benjamini-Hochberg.**
