# Supplementary material for: Association of NDRG4 gene methylation in peripheral blood leukocytes with gastric cancer risk, chemotherapy efficacy and prognosis
Source: Front Oncol. 2026 Apr 27;16:1778070. doi: 10.3389/fonc.2026.1778070 (PMC13158064; doi:10.3389/fonc.2026.1778070)

**Supplementary materials**

**Estimation of sample size**

The study enrolled 30 GC patients and 30 HCs, conducting a preliminary analysis of the association between the methylation level of NDRG4 gene and GC risk. Preliminary results showed that methylation of NDRG4-chr16:58497239 in PBLs was associated with GC risk. To ensure the scientific validity and methodological rigor of this study, the sample size was estimated based on preliminary data. The mean ± standard deviation was 0.75 ± 0.40 in the HCs group, and 0.99 ± 0.47 in the GC group. Sample size was estimated using a two-sample mean comparison approach. The methylation data showed skewed distribution, we applied nonparametric correction to adjust the estimated sample size (the formula was shown below). With *α* = 0.05 (two-tailed) and *β* = 0.1, the calculated required sample size was approximately 75 subjects for the GC group and the HCs group.


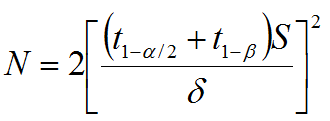

Supplement: Supplementary file 16 [file Table11.docx]
